# Diabetes and intervertebral disc degeneration: A Mendelian randomization study

**DOI:** 10.3389/fendo.2023.1100874

**Published:** 2023-02-28

**Authors:** Peihao Jin, Yonggang Xing, Bin Xiao, Yi Wei, Kai Yan, Jingwei Zhao, Wei Tian

**Affiliations:** Department of Spine Surgery, Beijing Jishuitan Hospital, Beijing, China

**Keywords:** type 2 diabetes mellitus, intervertebral disc degeneration, Mendelian randomization (MR) analysis, GWAS data, body mass index

## Abstract

**Introduction:**

Intervertebral disc degeneration (IVDD) is an important contributor of low back pain, which represents one of the most disabling symptoms within the adult population. Recently, increasing evidence suggests the potential association between Type 2 diabetes mellitus (T2DM) and IVDD. However, the causal relationship between these two common diseases remains unclear.

**Methods:**

We conducted a two-sample Mendelian randomization (MR) analysis to assess the causal association between T2DM and IVDD. Sensitivity analysis was performed to test for heterogeneity and horizontal pleiotropy. Multivariable MR was also conducted to adjust for the effect of BMI on IVDD.

**Results:**

A total of 128 independent single-nucleotide polymorphisms (SNPs) that were significantly associated with T2DM were selected as instrumental variables in univariable MR analysis. Our results showed that patients with T2DM had a higher risk of developing IVDD (OR, 1.069; 95% CI, 1.026–1.115; *p* = 0.002). The relationship remained stable in sensitive analysis including multivariable MR, which implicated the direct causal effect of T2DM on IVDD (OR, 1.080; 95% CI, 1.041–1.121; *p* < 0.001) after adjusting for BMI.

**Conclusions:**

MR analysis indicated a causal effect of T2DM on IVDD, and the effect persisted even when we accounted for the impact of BMI.

## Introduction

Intervertebral disc degeneration (IVDD) is currently a common degraded condition in an aging society, referring to an age-dependent, cell-mediated molecular process (1, 2). Degenerated discs are more prone to out-pouching and may press against the nerve roots, which eventually causes low back pain (LBP) or other clinical symptoms. As an increasingly prevalent health problem, IVDD significantly impacts patients’ quality of life and poses a substantial economic burden to countries with rapidly aging populations, such as China (3, 4). To date, in spite of the high prevalence of IVDD, lines of evidence for the risk factors of IVDD have not been fully established yet. Traditionally, IVDD is considered to be a multifactorial disease affected by both genetic and environmental factors including diabetes, obesity, and smoking ^5.^ Recently, increasing evidence has suggested that metabolic disturbances and inflammation might be involved in the development of IVDD, which shifts the focus of research to metabolic risk factors (5).

As the most common metabolic disorder, Type 2 diabetes mellitus (T2DM) threatened aging populations because of its various complications. Apart from being a strong risk factor for cardiovascular diseases and stroke, T2DM may also increase the risk of developing IVDD. To date, the potential relationship between diabetes and IVDD has been recognized in animal and clinical studies. In diabetic models, IVDD-related pathological changes in spine structure such as loss of disc height, decreased vertebral bone mass, and endplate sclerosis were well documented (6–9).However, in contrast to the consistently positive lines of evidence in laboratory studies, clinical studies have produced inconsistent results. Some researchers have inferred that T2DM is a significant risk factor for IVDD using cross-sectional and retrospective studies (10–13). Nevertheless, these cross-sectional or case–control studies failed to examine the independent association between DM and IVDD. It has been challenged that the correlation would disappear when controlling for body mass index (BMI) or other risk factors (14, 15). In fact, these inconsistent results may be due to the limitation of observational studies with susceptibility to bias and an inability to make causal inference.

Recently, Mendelian randomization (MR) studies, which use an epidemiological approach that assesses the causal effect of a risk factor on an outcome, have been increasingly used to overcome the aforementioned limitations and explore causal relationships (16). Since genetic variants are randomly assigned, the confounding factors are minimized by the MR method. Genetic variation significantly associated with exposure can therefore be used as instrumental variables (IVs). There are three assumptions that must be satisfied for instrumental variables: IV1, associated with the exposure; IV2, independent of the outcome given the exposure; and IV3, independent of all confounders known thus far ^16.^ To date, limited evidence for causal factors of IVDD has been reported. In particular, the relationship between diabetes and IVDD has not been fully investigated by MR.

In this regard, we explored the causal effect of T2DM on IVDD using a two-sample MR analysis. Furthermore, as BMI and T2DM are strongly correlated, and because previous observational studies and MR analysis have suggested that causal association may exist between BMI and IVDD (10, 13, 17–19), we therefore conducted a multivariable MR to examine the direct effect of T2DM on IVDD after adjusting for BMI.

## Materials and methods

### Study design

**Figure 1:**
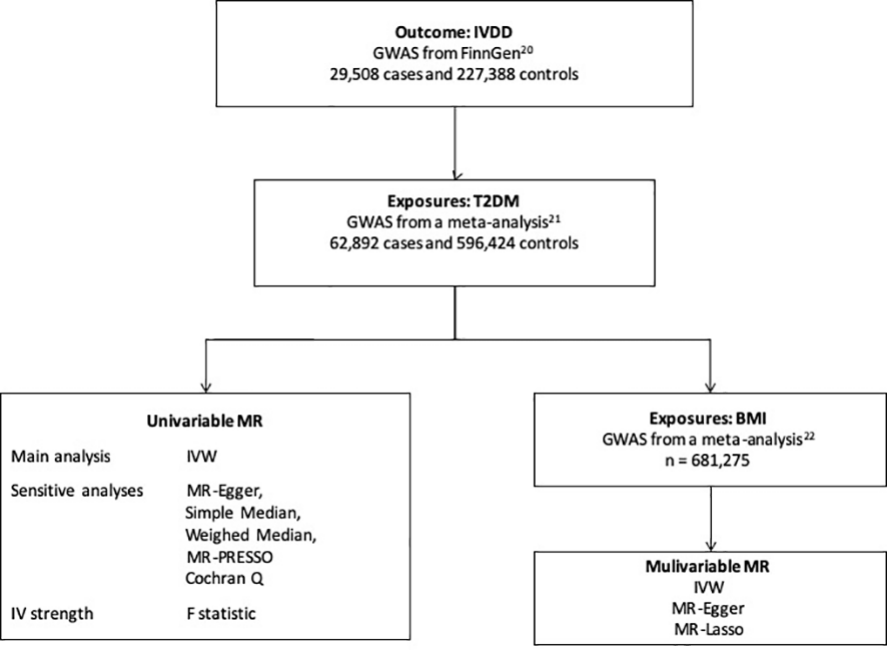
The study design. IVDD, intervertebral disc degeneration; T2DM, Type 2 diabetes mellitus; GWAS, genome-wide association study; MR, Mendelian randomization; BMI, body mass index; IVW, inverse-variance weighted.

The study design is shown in **Figure 1**. We first performed univariable MR to assess the causal relationship between T2DM and IVDD. Then, multivariable MR was conducted to adjust for BMI, which has been suggested to have a causal effect on IVDD (10, 13, 17–19), in order to assess the direct effect of T2DM. We used publicly available GWAS data with the informed consent and ethical approval previously obtained (20–22).

### GWAS data source

Summary GWAS data for IVDD were available from the FinnGen consortium, including 29,508 cases and 227,388 controls (20). IVDD was diagnosed according to ICD-10 M51, ICD-9 722, and ICD-8 275. Other detailed information of the outcome is presented in **Supplementary Table 1**.

The GWAS data of T2DM were from a meta-analysis with ~16 million genetic variants in 62,892 T2DM cases and 596,424 controls of European ancestry (21). Analysis was adjusted for age, sex, and the first 20 PCs. Genetic instruments for BMI were identified using results from the largest available meta-analysis of GWAS in 681,275 individuals of European ancestry (22).

### Instrumental variable selection

For univariable MR analysis, we first identified independent (linkage disequilibrium, LD clumping *r* ^2^ threshold = 0.001 and window size = 1,000 kb), genome-wide single-nucleotide polymorphisms (SNPs) significantly associated with T2DM (*p* < 5 × 10^−8^). For the multivariable MR analysis, we pooled all genome-wide significant SNPs that were significantly associated with T2DM or BMI and then clumped these SNPs with respect to the lowest *p*-value corresponding to any of the two using a 1,000-kb window and pairwise LD *r* ^2^ < 0.001. We calculated the proportion of phenotypic variance explained by instrumental variable SNPs of T2DM and computed the *F* statistic to verify whether they were strong instruments (23).

### MR analysis

We used the inverse-variance weighted (IVW) method as the primary MR approach (16). MR-Egger, weighted median, simple median tests, and MR-PRESSO were further conducted to control horizontal pleiotropy (16). We also used the Cochran *Q* statistic and MR-Egger (intercept) to test for the heterogeneity and pleiotropy (16).

Next, as BMI and T2DM are strongly correlated and the causal association may exist between BMI and IVDD in previous studies (10, 13, 17–19), we conducted multivariable MR adjusting for BMI to show the casual effect of T2DM on IVDD. The methods we used to conduct multivariable MR included IVW, MR-Egger, and MR-Lasso (24). Moreover, Cochran *Q* statistic and MR-Egger (intercept) were also conducted for the heterogeneity and pleiotropy of multivariable MR analysis. All statistical analyses were conducted using the “Two Sample MR” (version 0.5.6) and “Mendelian Randomization” (version 0.5.1) in the statistical program R (version 4.1.1). *p* < 0.05 was considered as statistically significant.

## Results

### Genetic instruments

We finally identified 128 independent SNPs that are significantly related to T2DM as instrumental variables (**Supplementary Table 2**). The phenotypic variances they accounted for was 13.9%, calculated by *R* ^2^. The *F* statistics of each SNP was greater than 10 (**Supplementary Table 2**). These findings suggested that there is no potential weak instrument bias, satisfying hypothesis IV1.

### Causal relationship between IVDD and T2DM

**Figure 2:**
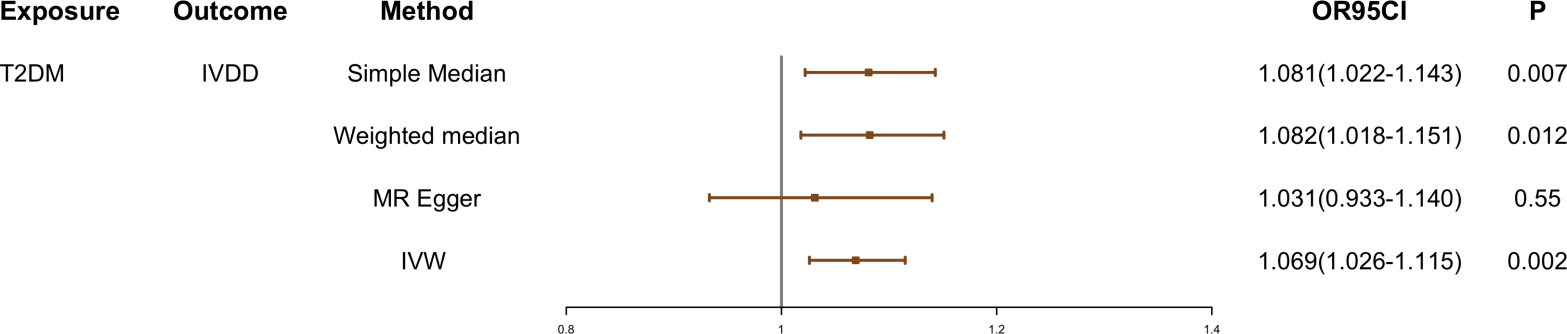
The causal effect of T2DM on IVDD. IVDD, intervertebral disc degeneration; T2DM, Type 2 diabetes mellitus; IVW, inverse-variance weighted. OR, odds ratio; CI, confidence interval.

Cochran *Q* test showed that there was instrumental heterogeneity (*p* < 0.05) (**Table 1**). Therefore, we employed the random-effect IVW method. The result showed that patients with T2DM have a 6.9% higher risk of IVDD than those without T2DM (OR, 1.069; 95% CI, 1.026–1.115; *p* = 0.002) (**Figure 2**).

**Table 1:** Results of Cochran *Q* test and MR-Egger intercept.

			IVW-Q test		MR-Egger		
Method	Outcome	Exposure	*Q* statistic	Q_p	Intercept	SE	*p*-value
Univariable MR	IVDD	T2DM	198.340	<0.001	0.003	0.004	0.435
Multivariable MR	IVDD	T2DM BMI	1,041.82	<0.001	<0.001	0.001	0.950

IVDD, intervertebral disc degeneration; T2DM, Type 2 diabetes mellitus; IVW: inverse-variance weighted, SE: standard error.

### Sensitivity analysis

**Figure 3:**
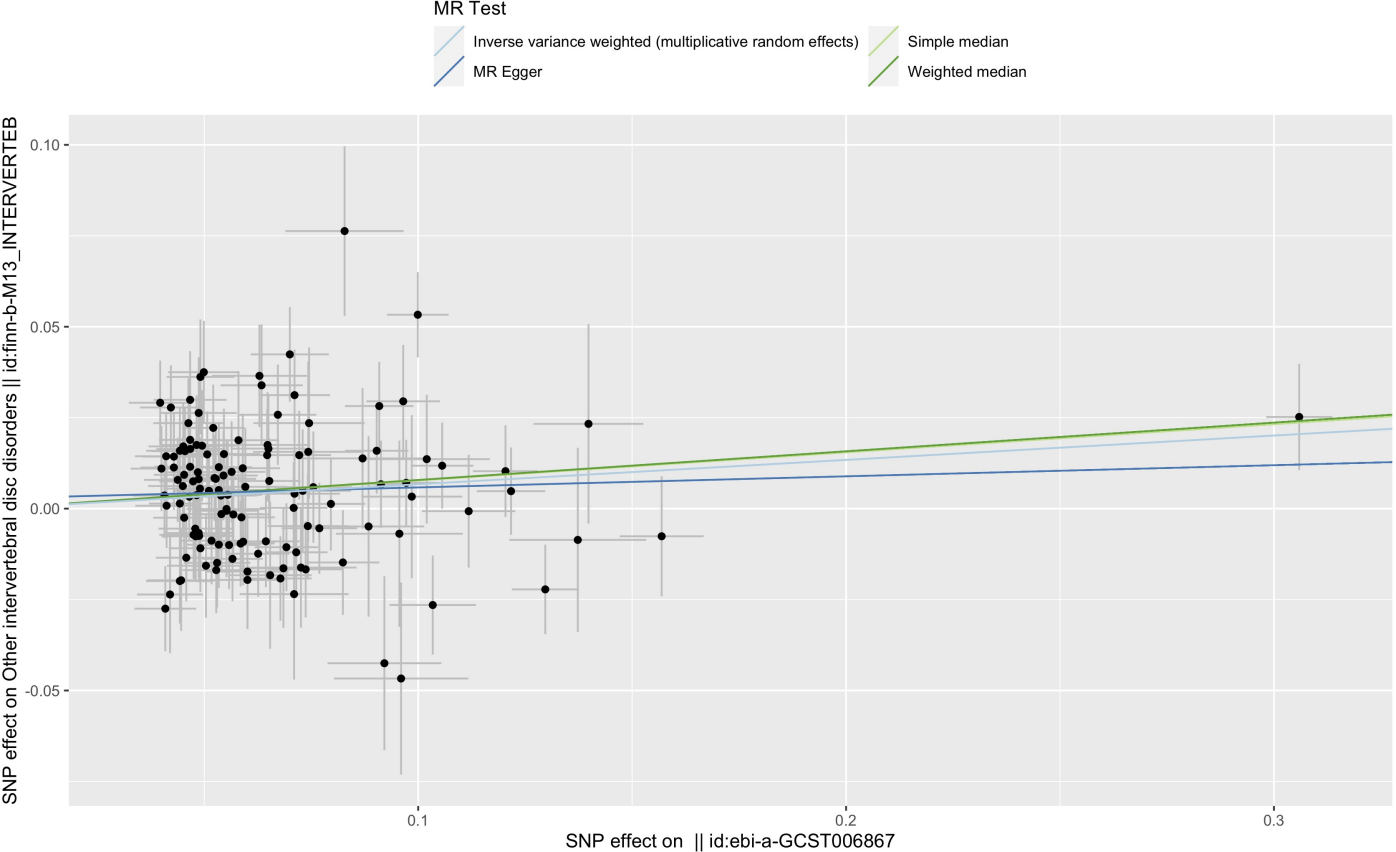
Scatter plot of the relationship between T2DM and IVDD using inverse-variance weighted, simple median, MR-Egger, and weighted median. SNP, single-nucleotide polymorphism; ebi-a-GCST006867, the GWAS ID of BMI; finn-b-M13-INTERVERTEB, the GWAS ID of IVDD.

The effect values obtained from simple median (OR, 1.081; 95% CI, 1.022–1.143; *p* = 0.007), weighted median (OR, 1.082; 95% CI, 1.018–1.151; *p* = 0.012), and MR-Egger (OR, 1.031; 95% CI, 0.933–1.140; *p* = 0.550) methods were consistent with the IVW estimate (**Figures 2** and **3**). There was also no significant differences between MR-Egger intercept and 0 (**Table 1**), which suggested no interference of horizontal pleiotropy in our study.

Furthermore, using MR-PRESSO, three outliers with horizontal pleiotropy were found. After removing these abnormal SNPs, we obtained corrected effect estimate showing similar results (OR, 1.059; 95% CI, 1.017–1.102; *p* = 0.006). The leave-one-out plot also showed that removing any of the SNPs did not change the results significantly, suggesting the reliability of the results (**Supplementary Figure 1**).

**Figure 4:**
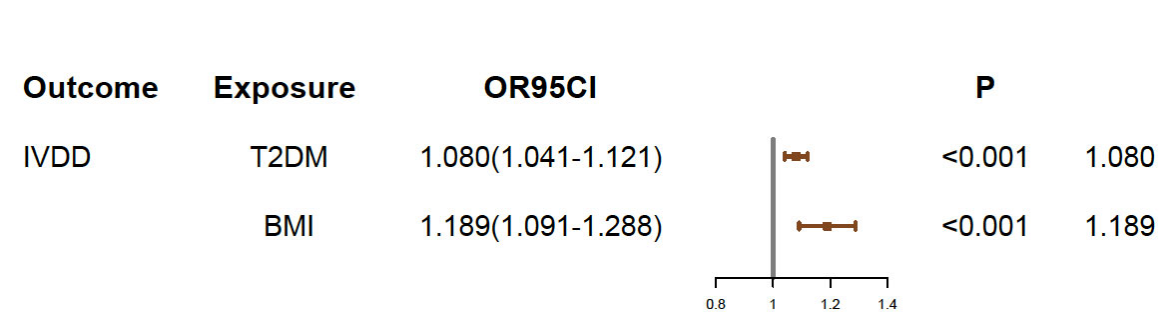
Multivariable MR results. IVDD, intervertebral disc degeneration; T2DM, Type 2 diabetes mellitus; OR, odds ratio; CI, confidence interval.

The causal association between BMI and IVDD was suggested in previous studies. Therefore, we conducted a multivariable MR analysis including both BMI and T2DM as exposures to explore the direct effect of T2DM on IVDD. There were 829 independent SNPs selected as instrumental variables for T2DM and BMI (**Supplementary Table 3**). Although the relationship between BMI and IVDD was confirmed (OR, 1.189; 95% CI, 1.091–1.288; *p* < 0.001), T2DM still showed a direct effect on IVDD (OR, 1.080; 95% CI, 1.041–1.121; p<0.001) conditioned on BMI (**Figure 4**) (**Table 2**). Moreover, multivariable MR-Egger suggested that there was no horizontal pleiotropy in MR analysis (Intercept *p* > 0.05). Moreover, although the Cochran *Q* test suggested that there may be heterogeneity (*p* < 0.01), the result of the MR-Egger test was the same as that of IVW (OR, 1.080; 95% CI, 1.041–1.121; *p* < 0.001) (**Table 2**). The result of the MR-Lasso test also remained stable after removing heterogeneous SNPs (OR, 1.078; 95% CI, 1.039–1.115; *p* < 0.001) (**Table 2**). Taken together, our results are proven to be reliable.

**Table 2:** Results of multivariable MR analysis.

MVMR Method	Exposure	Outcome	OR	95% CI	*p*
IVW	BMI	IVDD	1.189	1.097–1.288	<0.001
	T2DM		1.080	1.041–1.121	<0.001
MR-Egger	BMI	IVDD	1.183	0.980–1.428	0.08
	T2DM		1.080	1.041–1.121	<0.001
MR-Lasso	BMI	IVDD	1.190	1.105–1.282	<0.001
	T2DM		1.078	1.039–1.115	<0.001

MVMR, multivariable Mendelian randomization; IVDD, intervertebral disc degeneration; T2DM, Type 2 diabetes mellitus; BMI, body mass index; IVW, inverse-variance weighted; OR, odd ratio; CI, confidence interval.

## Discussion

To date, as the role of metabolic characteristics was evident in the development of IVDD, the influence of diabetes on IVDD has aroused widespread attention. However, strong clinical evidence for the direct relationship between diabetes and IVDD remains insufficient. In this study, we demonstrated that T2DM was an important risk factor causally associated with IVDD by using MR analysis. Furthermore, the multivariable MR suggested that the causal association between T2DM and IVDD was independent of BMI.

Our study was in line with five recent studies, which implicated the potential association between diabetes and IVDD. In 2016, Agius et al. first conducted a cross-sectional study on 100 patients with diabetes, investigating the changes in intervertebral disc of patients with T2DM. They found that diabetes might be a risk factor for IVDD since it is associated with significantly lower height of lumbar discs (11). Furthermore, a retrospective single-center study in Chinese patients with diabetes suggested that longer duration and poorly controlled T2DM were risk factors for lumbar disc degeneration. In addition, long-standing diabetes may be a predictor for severe IVDD (*p* < 0.05) (12).

Considering that the above samples were sill not sufficient enough, larger populations are required for adequate power. Hence, Jakoi et al. performed a cross-sectional study using a large insurance industry database in USA and discovered that IVDD is correlated with diabetes (10). Similarly, a case–control study that enrolled 160,911 patients with IVDD and 315,225 controls in a group of military members also suggested that diabetes was a risk factor for developing IVDD (13). However, these two large studies still have some limitations such as coding bias. Additionally, since the use of cross-sectional study design cannot confirm the causal relationship, Teraguchi et al. conducted the Wakayama Spine Study in a longitudinal population-based cohort, demonstrating that diabetes was a significant contributor to IVDD in the upper lumbar spine (OR, 6.83; 95% CI, 1.07-133.7) (25). However, these results should also be interpreted with caution, as the sample size of patients with diabetes was small. Therefore, large-scale studies and highly persuasive lines of evidence are needed to further validate the causal relationship between diabetes and IVDD.

With respect to the underlying mechanism, crucial aspects of the linked pathogenesis of IVDD in T2DM are identified using animal models. In general, IVDD consists of three main components: the inner nucleus pulposus (NP), the outer annulus fibrosus (AF), and the cartilaginous endplates (CEPs), which anchor the disc to the adjacent vertebrae (26). In T2DM, irreversible formation and accumulation of advanced glycation end products (AGEs) due to hyperglycemia may result in pathophysiological changes in CEPs and contribute to undermining the nutrient supply, cell viability, matrix homeostasis, and biomechanical properties of the intervertebral disc, leading to structural weakening and, ultimately, IVDD. Interestingly, preclinical evidence from a study of a rat model suggests that T2DM compromises IVDD composition, ECM homeostasis, and biomechanical behavior changes, rather than obesity (6). In summary, diabetic models indicated that hyperglycemia could exert a direct effect on IVDD by multiple diabetic-related pathways (1, 5, 27, 28).

As mentioned above, observational studies could not provide insight into the causal relationship between diabetes and IVDD, even based on a larger sample scale. Furthermore, unmeasured confounding variables, reverse causality, and survival bias may fail to give strong evidence on the relationship of interest. Therefore, we conducted the first MR study of T2DM and IVDD to address this uncertainty. MR analysis used genetic variants as instrumental variables for causal inferences about the effect of modifiable exposures on health- and disease-related outcomes in the presence of unobserved confounding variables (29). In consequence, differences in the outcome can be credited to the difference in the risk factor if the genetic variants are not related to confounders (30).

It should be noted that increasing evidence suggested that higher BMI, especially being overweight or obese, is associated with the risk of IVDD (10, 13, 17–19). Moreover, as higher BMI is interrelated with T2DM (31), the confounding effect of BMI should be paid attention to when discussing the relationship between T2DM and IVDD. As a result, we included both T2DM and BMI in our multivariable MR analysis to explore whether the effect of T2DM on IVDD is independent of BMI. In our study, although the causal association between BMI and IVDD was observed, T2DM was still associated with a higher risk of IVDD after adjusting for BMI in IVW analysis. Hence, we suggested that the causal effect of T2DM on IVDD persisted even when the impact of BMI was accounted for. In addition, the effect of BMI should also be considered when discussing other risk factors for IVDD.

The strengths of the study are as follows: First, a causal association was demonstrated using two large GWAS summary datasets for the first time, which is important for the prevention of IVDD and future clinical research. Second, we used multiple methods to test and account for heterogeneity and horizontal pleiotropy, in order to ensure the reliability of the results. Last, we used multivariable MR to examine the direct effect of T2DM on IVDD adjusting for BMI. However, some limitations should be noted: The GWAS data we used were from the European descent population, and the result cannot be generalized to other populations.

In summary, this is the first MR study to explore the causal effect of T2DM on IVDD, and the effect persisted even when we accounted for the impact of BMI. Moreover, further research is warranted to understand the biological mechanism of this causal effect.

## Data availability statement

The datasets presented in this study can be found in online repositories. The names of the repository/repositories and accession number(s) can be found in the article/**Supplementary Material**.

## Author contributions

PJ and WT conceptualized and designed the study. PJ, YX, BX, YW, KY, and JZ performed data analysis. PJ wrote the manuscript. All authors contributed to the article and approved the submitted version.
