# Supplementary material for: Diabetes and intervertebral disc degeneration: A Mendelian randomization study
Source: Front Endocrinol (Lausanne). 2023 Feb 28;14:1100874. doi: 10.3389/fendo.2023.1100874 (PMC10011653; doi:10.3389/fendo.2023.1100874)
Supplement: Supplementary file 1 [file DataSheet_1.docx]

# Supplementary Tables

Supplementary Table 1. Outcome information

| **Outcome** | **Consortium** | **ID** | **Cases** | **Control** | **Population** | **Definition/inclusion criteria** | **Adjustments** |
| --- | --- | --- | --- | --- | --- | --- | --- |
| IVDD | Finngen (R7) | M13_INTERVERTEB | 29508 | 227388 | European | ICD-10M51, ICD-9 722, ICD-8 725; excluded ICD-9 7220\|7224\|7227\|7228Av, ICD-87250 | sex, age, 10 PCs, genotyping batch |

Detailed information about ancestry assignment, cohort description, genotyping/imputation and association analyses can be obtained from Finngen consortium website (https://finngen.gitbook.io/documentation/releases)

Supplementary Table 2. Instrument variables.

| Chr | SNP | A1 | A2 | Beta | EAF | SE | p | R2 | F |
| --- | --- | --- | --- | --- | --- | --- | --- | --- | --- |
| 1 | rs1127655 | T | C | -0.0438 | 0.529064 | 0.0079 | 2.47E-08 | 0.000494 | 30.7393 |
| 1 | rs12088739 | G | A | -0.0884 | 0.089848 | 0.013 | 9.79E-12 | 0.000737 | 46.24 |
| 1 | rs2296173 | G | A | 0.065 | 0.212011 | 0.0087 | 7.66E-14 | 0.000888 | 55.81979 |
| 1 | rs2493394 | G | A | 0.073 | 0.107338 | 0.0113 | 1.15E-10 | 0.00066 | 41.73389 |
| 1 | rs2820426 | G | A | 0.0521 | 0.610058 | 0.0073 | 1.30E-12 | 0.0008 | 50.93657 |
| 1 | rs340874 | C | T | 0.0626 | 0.563904 | 0.0073 | 8.41E-18 | 0.001174 | 73.5365 |
| 1 | rs348330 | A | G | -0.0487 | 0.633451 | 0.0081 | 1.86E-09 | 0.000574 | 36.1483 |
| 2 | rs1009358 | C | T | -0.0545 | 0.377278 | 0.008 | 9.81E-12 | 0.000737 | 46.41016 |
| 2 | rs10169613 | T | C | -0.0429 | 0.472134 | 0.0078 | 3.57E-08 | 0.000483 | 30.25 |
| 2 | rs12617659 | T | C | -0.0685 | 0.147238 | 0.0103 | 2.83E-11 | 0.000704 | 44.22896 |
| 2 | rs13389219 | T | C | -0.0722 | 0.394368 | 0.0074 | 2.11E-22 | 0.001506 | 95.1943 |
| 2 | rs17334919 | T | C | -0.1398 | 0.100218 | 0.0128 | 6.69E-28 | 0.001905 | 119.2874 |
| 2 | rs243019 | C | T | 0.0566 | 0.455831 | 0.0071 | 2.29E-15 | 0.000998 | 63.55009 |
| 2 | rs2867125 | C | T | 0.0601 | 0.827825 | 0.0096 | 4.33E-10 | 0.000619 | 39.19282 |
| 2 | rs2972144 | G | A | 0.0913 | 0.645367 | 0.0075 | 2.55E-34 | 0.00237 | 148.19 |
| 2 | rs7561798 | G | A | 0.04 | 0.482171 | 0.0072 | 2.79E-08 | 0.00049 | 30.8642 |
| 2 | rs7572970 | G | A | 0.059 | 0.722047 | 0.0087 | 1.39E-11 | 0.000726 | 45.99022 |
| 2 | rs780094 | C | T | 0.0692 | 0.612845 | 0.0074 | 5.16E-21 | 0.001406 | 87.44777 |
| 3 | rs11708067 | G | A | -0.0965 | 0.23899 | 0.0086 | 5.93E-29 | 0.001981 | 125.9093 |
| 3 | rs11925227 | A | G | -0.0534 | 0.183439 | 0.0095 | 2.25E-08 | 0.000497 | 31.59623 |
| 3 | rs11926707 | C | T | 0.0463 | 0.625556 | 0.0082 | 1.69E-08 | 0.000506 | 31.88117 |
| 3 | rs1496653 | G | A | -0.0769 | 0.204782 | 0.0088 | 2.57E-18 | 0.001211 | 76.36377 |
| 3 | rs1899951 | T | C | -0.1118 | 0.123288 | 0.0109 | 1.64E-24 | 0.001659 | 105.2036 |
| 3 | rs2292662 | T | C | -0.0629 | 0.151272 | 0.0111 | 1.24E-08 | 0.000516 | 32.11111 |
| 3 | rs3887925 | T | C | 0.0474 | 0.553172 | 0.0079 | 2.47E-09 | 0.000565 | 36 |
| 3 | rs4472028 | C | T | -0.0453 | 0.55575 | 0.0071 | 2.08E-10 | 0.000642 | 40.70799 |
| 3 | rs6795735 | T | C | -0.0558 | 0.410912 | 0.0073 | 1.63E-14 | 0.000937 | 58.42822 |
| 3 | rs6808574 | C | T | 0.0552 | 0.609626 | 0.0076 | 4.39E-13 | 0.000834 | 52.75346 |
| 3 | rs7651090 | G | A | 0.1204 | 0.313377 | 0.0076 | 3.85E-57 | 0.004028 | 250.9723 |
| 3 | rs9844972 | C | G | 0.0956 | 0.069723 | 0.0148 | 1.03E-10 | 0.000664 | 41.72462 |
| 4 | rs11098676 | C | T | 0.054 | 0.787639 | 0.0096 | 2.03E-08 | 0.0005 | 31.64063 |
| 4 | rs17086692 | T | G | -0.0467 | 0.313426 | 0.0084 | 2.48E-08 | 0.000494 | 30.9083 |
| 4 | rs1801214 | T | C | 0.0903 | 0.599585 | 0.0074 | 5.52E-34 | 0.002346 | 148.906 |
| 4 | rs735949 | C | T | -0.0711 | 0.14115 | 0.0106 | 1.95E-11 | 0.000716 | 44.99119 |
| 4 | rs7674212 | T | G | -0.0465 | 0.408864 | 0.0075 | 6.18E-10 | 0.000608 | 38.44 |
| 4 | rs7685296 | T | C | -0.0511 | 0.279365 | 0.0081 | 2.32E-10 | 0.000639 | 39.79896 |
| 4 | rs993380 | G | A | -0.0507 | 0.66555 | 0.0081 | 4.59E-10 | 0.000617 | 39.17833 |
| 5 | rs10077431 | A | C | -0.0487 | 0.214668 | 0.0089 | 4.75E-08 | 0.000474 | 29.9418 |
| 5 | rs1061813 | A | G | -0.0429 | 0.537119 | 0.0073 | 3.37E-09 | 0.000556 | 34.53575 |
| 5 | rs2307111 | C | T | -0.0407 | 0.39727 | 0.0074 | 3.03E-08 | 0.000488 | 30.25 |
| 5 | rs459193 | G | A | 0.0711 | 0.745338 | 0.0083 | 8.81E-18 | 0.001172 | 73.3809 |
| 5 | rs4865796 | A | G | 0.053 | 0.69309 | 0.0078 | 1.33E-11 | 0.000728 | 46.17028 |
| 5 | rs6878122 | A | G | -0.0564 | 0.681791 | 0.0079 | 1.19E-12 | 0.000803 | 50.96876 |
| 5 | rs7729395 | T | C | 0.1373 | 0.050941 | 0.016 | 1.10E-17 | 0.001165 | 73.63785 |
| 6 | rs1050226 | G | A | -0.0491 | 0.406792 | 0.0074 | 3.34E-11 | 0.000699 | 44.02502 |
| 6 | rs1063355 | G | T | 0.0709 | 0.602436 | 0.0079 | 3.72E-19 | 0.001272 | 80.54494 |
| 6 | rs2246012 | C | T | 0.0527 | 0.164823 | 0.0094 | 2.43E-08 | 0.000495 | 31.43153 |
| 6 | rs2857605 | T | C | 0.0672 | 0.789389 | 0.009 | 5.90E-14 | 0.000896 | 55.75111 |
| 6 | rs622217 | C | T | -0.0485 | 0.483932 | 0.0077 | 3.13E-10 | 0.000629 | 39.67364 |
| 6 | rs72892910 | T | G | 0.0648 | 0.172394 | 0.0099 | 6.43E-11 | 0.000679 | 42.84298 |
| 6 | rs7756992 | G | A | 0.1297 | 0.266896 | 0.0078 | 6.00E-62 | 0.004377 | 276.4972 |
| 6 | rs853974 | C | T | -0.0601 | 0.737586 | 0.0088 | 7.86E-12 | 0.000744 | 46.64269 |
| 6 | rs9369425 | A | G | -0.0546 | 0.708185 | 0.0085 | 1.13E-10 | 0.000661 | 41.26173 |
| 7 | rs13239186 | T | C | 0.0539 | 0.302029 | 0.0085 | 2.70E-10 | 0.000634 | 40.21052 |
| 7 | rs17168486 | T | C | 0.0742 | 0.173603 | 0.0094 | 2.18E-15 | 0.001 | 62.30919 |
| 7 | rs2191348 | T | G | 0.0652 | 0.546935 | 0.0073 | 3.44E-19 | 0.001274 | 79.77181 |
| 7 | rs2299383 | T | C | 0.0412 | 0.423455 | 0.0073 | 1.49E-08 | 0.00051 | 31.85288 |
| 7 | rs2908282 | A | G | 0.0552 | 0.177395 | 0.0094 | 4.25E-09 | 0.000549 | 34.48438 |
| 7 | rs7786095 | G | A | -0.0743 | 0.10386 | 0.0129 | 9.64E-09 | 0.000523 | 33.17403 |
| 7 | rs849135 | A | G | -0.0999 | 0.499052 | 0.0072 | 1.04E-43 | 0.003052 | 192.5156 |
| 8 | rs10087241 | A | G | -0.0475 | 0.594893 | 0.008 | 2.80E-09 | 0.000561 | 35.25391 |
| 8 | rs10100265 | C | A | -0.0491 | 0.61049 | 0.0079 | 6.29E-10 | 0.000608 | 38.62859 |
| 8 | rs11774915 | T | C | 0.0495 | 0.337129 | 0.0086 | 8.73E-09 | 0.000526 | 33.12939 |
| 8 | rs12681990 | C | T | 0.0634 | 0.16373 | 0.0096 | 3.62E-11 | 0.000696 | 43.61502 |
| 8 | rs17411031 | G | C | -0.045 | 0.261729 | 0.0081 | 3.04E-08 | 0.000488 | 30.8642 |
| 8 | rs2294120 | G | A | -0.0443 | 0.455879 | 0.0079 | 1.62E-08 | 0.000507 | 31.44512 |
| 8 | rs3802177 | A | G | -0.1217 | 0.311281 | 0.008 | 2.32E-52 | 0.00368 | 231.4202 |
| 8 | rs516946 | C | T | 0.0824 | 0.760633 | 0.0085 | 3.16E-22 | 0.001494 | 93.97592 |
| 8 | rs7841082 | T | C | -0.042 | 0.438276 | 0.0077 | 4.94E-08 | 0.000473 | 29.75207 |
| 8 | rs7845219 | C | T | -0.0422 | 0.492786 | 0.0072 | 4.54E-09 | 0.000546 | 34.35262 |
| 9 | rs10114341 | C | T | -0.0409 | 0.440754 | 0.0072 | 1.15E-08 | 0.000518 | 32.26871 |
| 9 | rs10811661 | C | T | -0.1569 | 0.173605 | 0.0098 | 4.13E-58 | 0.004098 | 256.3266 |
| 9 | rs10974438 | C | A | 0.0591 | 0.351215 | 0.0075 | 3.01E-15 | 0.000989 | 62.0944 |
| 9 | rs1333039 | C | G | 0.0534 | 0.598788 | 0.0074 | 5.64E-13 | 0.000826 | 52.07378 |
| 9 | rs17791483 | G | A | -0.102 | 0.062588 | 0.0147 | 3.42E-12 | 0.00077 | 48.14661 |
| 9 | rs2796441 | A | G | -0.0715 | 0.416458 | 0.0073 | 1.96E-22 | 0.001509 | 95.93263 |
| 10 | rs11257655 | T | C | 0.0737 | 0.206773 | 0.0087 | 1.97E-17 | 0.001147 | 71.76232 |
| 10 | rs2421016 | T | C | -0.0458 | 0.473761 | 0.0071 | 1.48E-10 | 0.000653 | 41.61159 |
| 10 | rs2616132 | A | G | 0.0455 | 0.474257 | 0.0078 | 6.58E-09 | 0.000535 | 34.02778 |
| 10 | rs2633310 | T | G | -0.0443 | 0.435073 | 0.0079 | 2.37E-08 | 0.000495 | 31.44512 |
| 10 | rs753270 | C | T | 0.0528 | 0.583535 | 0.0079 | 2.70E-11 | 0.000705 | 44.66976 |
| 10 | rs7903146 | T | C | 0.3059 | 0.291585 | 0.0077 | 1.00E-200 | 0.014425 | 1578.256 |
| 10 | rs7923866 | T | C | -0.0972 | 0.378775 | 0.0074 | 9.34E-40 | 0.002765 | 172.5318 |
| 11 | rs10830963 | G | C | 0.0909 | 0.275768 | 0.008 | 5.85E-30 | 0.002054 | 129.1064 |
| 11 | rs1552224 | C | A | -0.1034 | 0.15421 | 0.0101 | 8.64E-25 | 0.001679 | 104.8089 |
| 11 | rs2237892 | T | C | -0.096 | 0.06249 | 0.0157 | 8.75E-10 | 0.000597 | 37.38894 |
| 11 | rs5215 | T | C | -0.0678 | 0.639941 | 0.0073 | 2.09E-20 | 0.001362 | 86.26084 |
| 11 | rs67232546 | T | C | 0.0596 | 0.209222 | 0.0096 | 4.66E-10 | 0.000617 | 38.5434 |
| 11 | rs7929543 | C | A | 0.0828 | 0.08316 | 0.0138 | 2.20E-09 | 0.000569 | 36 |
| 12 | rs10842994 | T | C | -0.0755 | 0.197025 | 0.0091 | 1.02E-16 | 0.001096 | 68.83529 |
| 12 | rs11048456 | T | C | -0.0488 | 0.756179 | 0.0082 | 2.97E-09 | 0.00056 | 35.41701 |
| 12 | rs11107116 | T | G | 0.0467 | 0.219714 | 0.0085 | 3.75E-08 | 0.000481 | 30.18533 |
| 12 | rs12299509 | G | A | 0.0467 | 0.478622 | 0.0073 | 2.09E-10 | 0.000642 | 40.92494 |
| 12 | rs2261181 | T | C | 0.0985 | 0.09647 | 0.0118 | 9.18E-17 | 0.001099 | 69.68005 |
| 12 | rs61953351 | T | G | -0.07 | 0.249902 | 0.0091 | 1.98E-14 | 0.000931 | 59.1716 |
| 12 | rs7138300 | T | C | -0.0443 | 0.556835 | 0.0072 | 5.65E-10 | 0.000611 | 37.85667 |
| 12 | rs825476 | T | C | 0.0524 | 0.580549 | 0.0073 | 6.80E-13 | 0.00082 | 51.52486 |
| 12 | rs940904 | A | G | 0.0499 | 0.743373 | 0.0083 | 2.08E-09 | 0.000571 | 36.14472 |
| 13 | rs1359790 | A | G | -0.0796 | 0.2867 | 0.008 | 2.80E-23 | 0.00157 | 99.0025 |
| 13 | rs576674 | A | G | -0.0654 | 0.832515 | 0.0097 | 1.79E-11 | 0.000718 | 45.45818 |
| 13 | rs963740 | T | A | -0.0479 | 0.294299 | 0.0086 | 2.23E-08 | 0.000497 | 31.02231 |
| 14 | rs7144011 | T | G | 0.0482 | 0.221063 | 0.0085 | 1.64E-08 | 0.000507 | 32.15557 |
| 15 | rs12910825 | G | A | 0.0517 | 0.360391 | 0.0074 | 2.16E-12 | 0.000784 | 48.81099 |
| 15 | rs4502156 | C | T | -0.0411 | 0.435857 | 0.0073 | 1.66E-08 | 0.000506 | 31.69844 |
| 15 | rs4932143 | C | G | -0.0568 | 0.720944 | 0.0087 | 5.51E-11 | 0.000683 | 42.62439 |
| 15 | rs7177055 | A | G | 0.0647 | 0.718289 | 0.0079 | 2.75E-16 | 0.001064 | 67.07403 |
| 15 | rs982077 | G | A | -0.0453 | 0.565521 | 0.0072 | 2.58E-10 | 0.000635 | 39.58507 |
| 16 | rs244415 | A | G | -0.0467 | 0.412872 | 0.0079 | 3.88E-09 | 0.000551 | 34.94456 |
| 16 | rs2925979 | C | T | -0.0534 | 0.70085 | 0.0078 | 9.06E-12 | 0.000739 | 46.86982 |
| 16 | rs7185735 | G | A | 0.1056 | 0.397306 | 0.0073 | 1.59E-47 | 0.003329 | 209.258 |
| 16 | rs9940149 | A | G | -0.058 | 0.178556 | 0.0095 | 9.29E-10 | 0.000596 | 37.27424 |
| 17 | rs12945601 | C | T | -0.048 | 0.613603 | 0.008 | 1.72E-09 | 0.000577 | 36 |
| 17 | rs17405722 | A | G | 0.087 | 0.074153 | 0.0146 | 2.28E-09 | 0.000568 | 35.50854 |
| 17 | rs17631783 | T | C | -0.0487 | 0.26346 | 0.0089 | 3.95E-08 | 0.00048 | 29.9418 |
| 17 | rs302864 | A | G | 0.071 | 0.087026 | 0.0127 | 2.46E-08 | 0.000494 | 31.25426 |
| 17 | rs8068804 | A | G | 0.0587 | 0.325097 | 0.0078 | 4.41E-14 | 0.000906 | 56.63527 |
| 17 | rs9894220 | G | A | -0.0585 | 0.43374 | 0.0079 | 1.52E-13 | 0.000867 | 54.83496 |
| 17 | rs9911983 | T | C | 0.0397 | 0.566573 | 0.0073 | 4.82E-08 | 0.000474 | 29.57572 |
| 18 | rs12970134 | A | G | 0.0555 | 0.26512 | 0.008 | 5.31E-12 | 0.000756 | 48.12891 |
| 18 | rs7240767 | C | T | 0.0451 | 0.383677 | 0.0081 | 2.16E-08 | 0.000498 | 31.00152 |
| 19 | rs10401969 | C | T | 0.0921 | 0.076586 | 0.0133 | 4.13E-12 | 0.000764 | 47.95302 |
| 19 | rs8108269 | G | T | 0.0644 | 0.281015 | 0.0079 | 3.11E-16 | 0.001061 | 66.45345 |
| 20 | rs4810426 | T | C | 0.0726 | 0.096789 | 0.013 | 2.15E-08 | 0.000498 | 31.18793 |
| 20 | rs6059662 | G | A | 0.0446 | 0.663176 | 0.0079 | 1.51E-08 | 0.000509 | 31.87246 |
| 20 | rs6066138 | A | G | -0.049 | 0.278362 | 0.0082 | 1.93E-09 | 0.000573 | 35.70791 |
| 20 | rs6515236 | C | A | -0.0504 | 0.24933 | 0.0091 | 3.34E-08 | 0.000485 | 30.67456 |
| 22 | rs16988333 | G | A | -0.0745 | 0.090404 | 0.013 | 9.17E-09 | 0.000525 | 32.84172 |
| 22 | rs4823182 | G | A | 0.0482 | 0.335748 | 0.0077 | 3.36E-10 | 0.000627 | 39.18435 |

Chr, chromosome; SNP, single nucleotide polymorphism; A1, effect allele; A2, other allele; EAF, A1 allele frequency; SE, standard error.

Supplementary Table 3. Instrument variables in MVMR.

| SNP | DM_beta | BMI_beta | DM_se | BMI_se | outcome_beta | outcome_se |
| --- | --- | --- | --- | --- | --- | --- |
| rs10007906 | 0.0067 | 0.0133 | 0.0082 | 0.0018 | -3.00E-04 | 0.0123 |
| rs10009336 | -0.0192 | -0.014 | 0.0097 | 0.0022 | -0.0181 | 0.0157 |
| rs1000940 | 0.0181 | 0.0154 | 0.0079 | 0.0018 | 0.001 | 0.0118 |
| rs1006896 | -0.0292 | -0.0234 | 0.0126 | 0.0027 | -0.0283 | 0.0191 |
| rs10071816 | 0.0383 | 0.0123 | 0.0099 | 0.0022 | 0.0141 | 0.0155 |
| rs1007934 | -0.0117 | -0.012 | 0.008 | 0.0017 | -0.0146 | 0.0116 |
| rs1009358 | -0.0545 | -0.0036 | 0.008 | 0.0018 | -0.0091 | 0.0123 |
| rs10118866 | 0.0082 | 0.012 | 0.0094 | 0.0021 | 0.0046 | 0.0131 |
| rs10131645 | -0.0276 | -0.0132 | 0.0093 | 0.0021 | 0.0156 | 0.018 |
| rs10131761 | -0.0119 | -0.0157 | 0.01 | 0.0022 | 0.0033 | 0.0134 |
| rs10131890 | 0.0274 | 0.0222 | 0.0176 | 0.0039 | 0.0266 | 0.0183 |
| rs10132280 | -0.0374 | -0.0223 | 0.0079 | 0.0018 | -0.0117 | 0.0122 |
| rs10144318 | -0.0122 | -0.0092 | 0.0071 | 0.0016 | -0.0103 | 0.0119 |
| rs10163018 | -0.0143 | -0.0114 | 0.0087 | 0.0018 | -0.0019 | 0.0117 |
| rs10169613 | -0.0429 | 0.0055 | 0.0078 | 0.0017 | -0.0113 | 0.0117 |
| rs10182181 | 0.0086 | 0.0325 | 0.0072 | 0.0016 | -3.00E-04 | 0.0118 |
| rs10193760 | -0.0027 | -0.0103 | 0.0079 | 0.0018 | -0.0065 | 0.0116 |
| rs10197031 | 0.0211 | 0.0166 | 0.0087 | 0.0019 | -0.0049 | 0.0122 |
| rs10198345 | 0.0133 | 0.0109 | 0.0083 | 0.0018 | 0.0134 | 0.0124 |
| rs1020548 | -0.0062 | -0.0132 | 0.0104 | 0.0023 | -0.0093 | 0.0148 |
| rs1021066 | -7.00E-04 | 0.0096 | 0.0078 | 0.0017 | 0.0215 | 0.0123 |
| rs10211055 | 0.0275 | 0.0157 | 0.0082 | 0.0018 | 0.0312 | 0.0125 |
| rs10243319 | -0.0109 | -0.0107 | 0.008 | 0.0018 | 0.0149 | 0.0117 |
| rs10263780 | -0.0211 | -0.0157 | 0.0115 | 0.0027 | 0.0411 | 0.0174 |
| rs10269783 | 0.0128 | 0.0133 | 0.0074 | 0.0017 | 2.00E-04 | 0.0116 |
| rs1037587 | 0.007 | 0.0106 | 0.0079 | 0.0017 | 0.0088 | 0.0116 |
| rs1038088 | 0.0155 | 0.0117 | 0.0076 | 0.0016 | 0.0086 | 0.0116 |
| rs10425678 | -0.0102 | 0.0096 | 0.0079 | 0.0017 | -0.0024 | 0.0116 |
| rs10433609 | -0.0023 | 0.0162 | 0.0109 | 0.0023 | -0.0103 | 0.0157 |
| rs10435879 | -0.011 | -0.0097 | 0.0079 | 0.0017 | -0.0026 | 0.0122 |
| rs10460960 | 0.0306 | 0.0197 | 0.0113 | 0.0025 | 0.0188 | 0.0194 |
| rs1048303 | -0.013 | -0.0111 | 0.008 | 0.0018 | -0.0048 | 0.0124 |
| rs1048932 | -0.0183 | -0.016 | 0.0073 | 0.0017 | -0.0119 | 0.0117 |
| rs10497810 | -0.029 | -0.0167 | 0.0103 | 0.0022 | -0.0032 | 0.0143 |
| rs1050226 | -0.0491 | 1.00E-04 | 0.0074 | 0.0018 | 0.0109 | 0.012 |
| rs1061813 | -0.0429 | -0.0019 | 0.0073 | 0.0017 | -0.0143 | 0.0119 |
| rs10732321 | -0.0162 | -0.0145 | 0.0112 | 0.0025 | -0.0056 | 0.0189 |
| rs10733051 | -0.0108 | -0.0097 | 0.0072 | 0.0016 | -0.0077 | 0.0117 |
| rs10744146 | -0.0108 | -0.0122 | 0.0077 | 0.0017 | -0.0067 | 0.0116 |
| rs10745785 | -0.0097 | -0.0111 | 0.0083 | 0.0018 | 0.0279 | 0.0119 |
| rs10757831 | 0.0194 | 0.0123 | 0.0099 | 0.0022 | 0.009 | 0.0151 |
| rs1075901 | 0.0034 | 0.0121 | 0.0072 | 0.0016 | 0.0035 | 0.0118 |
| rs10760678 | -0.0247 | -0.0113 | 0.0079 | 0.0017 | 0.0044 | 0.0118 |
| rs10768994 | -0.0149 | -0.0114 | 0.0073 | 0.0017 | 0.0187 | 0.0118 |
| rs10772055 | 0.0496 | 0.0172 | 0.0105 | 0.0024 | -0.0144 | 0.0167 |
| rs10779751 | 0.0195 | -0.0139 | 0.0081 | 0.0018 | -0.0123 | 0.0133 |
| rs10795422 | 0.0263 | 0.0139 | 0.0085 | 0.0019 | 0.0084 | 0.013 |
| rs10797115 | 0.0119 | 0.0124 | 0.0079 | 0.0017 | 0.0165 | 0.0116 |
| rs10811661 | -0.1569 | 0.0065 | 0.0098 | 0.0022 | 0.0076 | 0.0165 |
| rs10811901 | 0.0271 | 0.0111 | 0.0073 | 0.0017 | -0.0155 | 0.0118 |
| rs10818938 | 0.0077 | 0.0114 | 0.0079 | 0.0017 | 0.0345 | 0.0117 |
| rs10830452 | 0.0056 | 0.0111 | 0.0083 | 0.0018 | 0.0194 | 0.0123 |
| rs10830963 | 0.0909 | 0.0075 | 0.008 | 0.0019 | 0.0282 | 0.0121 |
| rs10838465 | -0.0226 | -0.014 | 0.0084 | 0.0019 | 0.007 | 0.012 |
| rs10842994 | -0.0755 | 5.00E-04 | 0.0091 | 0.0021 | -0.0059 | 0.0153 |
| rs10878946 | -0.0231 | -0.0141 | 0.0087 | 0.0019 | -0.0036 | 0.0121 |
| rs10886017 | 0.0197 | 0.0152 | 0.0082 | 0.0019 | 0.0039 | 0.0135 |
| rs10915840 | -0.008 | -0.0118 | 0.0087 | 0.0019 | -0.0048 | 0.0138 |
| rs10920678 | -0.0095 | -0.0155 | 0.0072 | 0.0016 | -0.0072 | 0.0116 |
| rs10923724 | -0.0037 | -0.0118 | 0.0073 | 0.0016 | -0.023 | 0.0125 |
| rs10929925 | -0.03 | -0.0143 | 0.0073 | 0.0016 | -0.0122 | 0.0116 |
| rs10933952 | 0.0163 | 0.0107 | 0.0079 | 0.0017 | -0.0147 | 0.0117 |
| rs10935143 | -0.0133 | -0.0109 | 0.0078 | 0.0017 | 0.0079 | 0.0121 |
| rs10938397 | 0.0293 | 0.0324 | 0.0073 | 0.0016 | 0.0336 | 0.0116 |
| rs10939792 | 0.0255 | 0.0152 | 0.0084 | 0.0019 | 0.018 | 0.0133 |
| rs10942267 | -0.0051 | -0.0156 | 0.0085 | 0.0019 | -0.0342 | 0.0141 |
| rs10942476 | 0.0179 | -0.0099 | 0.0079 | 0.0017 | -0.0079 | 0.0117 |
| rs10951356 | 0.0208 | 0.0104 | 0.0087 | 0.0018 | -0.0022 | 0.012 |
| rs10962549 | 0.0222 | 0.0198 | 0.0103 | 0.0023 | 0.0338 | 0.0165 |
| rs10968114 | -0.0078 | -0.0113 | 0.0079 | 0.0017 | 0.0287 | 0.0116 |
| rs10971721 | -0.0245 | -0.0199 | 0.0127 | 0.0028 | 0.0347 | 0.015 |
| rs10974438 | 0.0591 | -0.0023 | 0.0075 | 0.0017 | -0.0091 | 0.012 |
| rs10989568 | -0.005 | -0.0107 | 0.0077 | 0.0017 | -0.0061 | 0.0116 |
| rs11001963 | 0.0186 | 0.0108 | 0.0082 | 0.0018 | 0.0162 | 0.0118 |
| rs11048456 | -0.0488 | 0.0054 | 0.0082 | 0.0019 | 0.0075 | 0.0128 |
| rs11056875 | -0.0244 | -0.0138 | 0.0109 | 0.0024 | -0.0076 | 0.0172 |
| rs11066188 | 0.0185 | -0.012 | 0.0074 | 0.0017 | -0.0033 | 0.0119 |
| rs11075489 | -0.0112 | -0.0111 | 0.0079 | 0.0017 | -0.0046 | 0.0117 |
| rs11084553 | -0.0396 | -0.021 | 0.011 | 0.0024 | 0.0432 | 0.0173 |
| rs11096549 | -3.00E-04 | 0.0109 | 0.0086 | 0.0019 | 0.0028 | 0.012 |
| rs11098676 | 0.054 | -0.0062 | 0.0096 | 0.0021 | -0.0015 | 0.0151 |
| rs11105839 | -0.0177 | -0.0109 | 0.0075 | 0.0017 | 0.0054 | 0.0121 |
| rs11107116 | 0.0467 | -0.0027 | 0.0085 | 0.002 | 0.0299 | 0.0134 |
| rs11115176 | -0.0243 | -0.0121 | 0.0084 | 0.0019 | -0.024 | 0.0139 |
| rs11121210 | 0.0092 | 0.0108 | 0.0082 | 0.0018 | -0.0138 | 0.0127 |
| rs11124991 | 0.0213 | 0.0123 | 0.0085 | 0.0018 | -0.0312 | 0.0122 |
| rs11128760 | -0.0302 | -0.011 | 0.0079 | 0.0017 | 0.0011 | 0.0119 |
| rs11128904 | 0.0247 | 0.0106 | 0.0088 | 0.0019 | -0.009 | 0.0123 |
| rs11129662 | 0.0324 | 0.0121 | 0.0083 | 0.0018 | 0.013 | 0.0128 |
| rs11138082 | -0.0013 | -0.0123 | 0.0096 | 0.0022 | -0.0209 | 0.0161 |
| rs11150911 | -0.0254 | -0.0133 | 0.008 | 0.0018 | 0.0117 | 0.0126 |
| rs11165643 | 0.0239 | 0.0206 | 0.0073 | 0.0017 | 0.0011 | 0.0118 |
| rs11170468 | -0.0114 | -0.0123 | 0.0086 | 0.0019 | -0.0096 | 0.0149 |
| rs1117080 | -0.0221 | -0.011 | 0.0086 | 0.0019 | 0.0025 | 0.0133 |
| rs11173522 | 0.0349 | 0.0128 | 0.0096 | 0.0021 | -0.0079 | 0.0147 |
| rs11208662 | 0.0594 | 0.0209 | 0.014 | 0.0031 | 0.0189 | 0.0207 |
| rs11223585 | -0.0121 | -0.0106 | 0.008 | 0.0018 | 0.0167 | 0.0126 |
| rs11228824 | 0.0095 | -0.0128 | 0.0103 | 0.0022 | 0.0012 | 0.0138 |
| rs11251352 | 0.0184 | 0.0109 | 0.0079 | 0.0018 | 0.0108 | 0.0116 |
| rs11257655 | 0.0737 | -0.0018 | 0.0087 | 0.002 | -0.0167 | 0.0132 |
| rs1125804 | -0.0215 | -0.0105 | 0.0079 | 0.0018 | -0.0082 | 0.0116 |
| rs1127655 | -0.0438 | 0.0013 | 0.0079 | 0.0017 | -0.0079 | 0.0116 |
| rs11496125 | 0.0383 | 0.0169 | 0.0079 | 0.0017 | 0.0014 | 0.0116 |
| rs1150659 | 0.0027 | -0.0139 | 0.0086 | 0.0019 | -0.0073 | 0.0143 |
| rs11538 | 0.0105 | 0.0135 | 0.0103 | 0.0023 | -0.0161 | 0.0147 |
| rs11577094 | -0.0017 | 0.0182 | 0.0131 | 0.003 | 0.0161 | 0.02 |
| rs1158684 | -0.0013 | 0.0099 | 0.0077 | 0.0017 | 0.0255 | 0.0119 |
| rs1158805 | -0.0159 | -0.0137 | 0.0081 | 0.0018 | -0.0028 | 0.0117 |
| rs11611246 | 0.0244 | 0.024 | 0.0096 | 0.002 | 0.0294 | 0.0142 |
| rs11635675 | 1.00E-04 | -0.0124 | 0.0081 | 0.0018 | 0.0092 | 0.0119 |
| rs11658335 | -0.0073 | -0.0101 | 0.0078 | 0.0017 | -0.0045 | 0.0117 |
| rs11670142 | 0.0093 | 0.0111 | 0.0081 | 0.0018 | -0.0149 | 0.0116 |
| rs11687101 | -0.0155 | -0.0133 | 0.0093 | 0.0021 | 0.0125 | 0.014 |
| rs11708067 | -0.0965 | 0.0078 | 0.0086 | 0.0019 | -0.0295 | 0.0155 |
| rs11708733 | -0.0533 | -0.0227 | 0.0185 | 0.0041 | -0.018 | 0.024 |
| rs11713193 | 0.0352 | 0.0239 | 0.008 | 0.0017 | 0.0099 | 0.0118 |
| rs11736228 | -0.0151 | -0.0139 | 0.009 | 0.002 | -0.0075 | 0.0138 |
| rs11738695 | 0.0051 | 0.0097 | 0.0079 | 0.0017 | 0.0022 | 0.0117 |
| rs11739877 | 0.0281 | 0.0117 | 0.0081 | 0.0018 | -0.0194 | 0.0116 |
| rs11754747 | -0.0014 | 0.0116 | 0.0083 | 0.0019 | -0.0218 | 0.0143 |
| rs11781222 | -0.0216 | -0.0158 | 0.0106 | 0.0024 | -0.0096 | 0.0206 |
| rs11784576 | -0.0016 | 0.0109 | 0.0086 | 0.0019 | 0.0092 | 0.0123 |
| rs11790280 | 0.0286 | 0.0103 | 0.0081 | 0.0018 | 0.0033 | 0.0119 |
| rs11792311 | -0.0091 | -0.0144 | 0.0093 | 0.002 | -0.0176 | 0.0133 |
| rs11847697 | 0.0576 | 0.0301 | 0.0177 | 0.004 | 0.0871 | 0.0477 |
| rs1187352 | -0.0057 | 0.0119 | 0.0081 | 0.0018 | -0.0147 | 0.0125 |
| rs11889536 | -0.0293 | -0.0189 | 0.0111 | 0.0024 | -0.0427 | 0.0265 |
| rs11904490 | -0.013 | -0.0099 | 0.0081 | 0.0018 | -0.0093 | 0.0123 |
| rs11915371 | 0.0314 | 0.0149 | 0.0096 | 0.0021 | -0.0126 | 0.0148 |
| rs11926707 | 0.0463 | -0.0028 | 0.0082 | 0.0018 | 0.0235 | 0.0123 |
| rs11945861 | -0.0069 | -0.0148 | 0.0093 | 0.002 | 0.0179 | 0.0139 |
| rs11951673 | -0.0055 | -0.0123 | 0.0074 | 0.0017 | -3.00E-04 | 0.0126 |
| rs1199334 | -0.0172 | -0.0142 | 0.0092 | 0.0021 | 0.0139 | 0.0142 |
| rs12022461 | -0.0347 | -0.0159 | 0.0104 | 0.0023 | -0.0171 | 0.0148 |
| rs12035349 | -0.0067 | 0.0188 | 0.0122 | 0.0026 | -0.0204 | 0.016 |
| rs12041258 | -0.023 | -0.0146 | 0.0094 | 0.002 | -0.0051 | 0.0136 |
| rs12044597 | -0.0044 | 0.0143 | 0.0073 | 0.0016 | -0.0037 | 0.0116 |
| rs12052672 | -0.0051 | -0.0112 | 0.0088 | 0.0019 | -0.033 | 0.0129 |
| rs12062845 | 0.0358 | 0.0139 | 0.0095 | 0.0021 | 0.0177 | 0.0127 |
| rs12065553 | 0.0213 | 0.0115 | 0.0085 | 0.0019 | 0.0199 | 0.0144 |
| rs12098284 | 0.0217 | 0.0178 | 0.0119 | 0.0026 | -0.0328 | 0.0224 |
| rs12101393 | -0.0284 | -0.0127 | 0.0096 | 0.0021 | 0.0108 | 0.0134 |
| rs12147845 | 0.045 | 0.0199 | 0.0125 | 0.0027 | 0.0379 | 0.0167 |
| rs12148330 | 0.0011 | -0.0142 | 0.0102 | 0.0022 | 0.0166 | 0.017 |
| rs12149756 | -0.0012 | -0.0125 | 0.0083 | 0.0019 | 0.0052 | 0.0135 |
| rs12150665 | -0.016 | -0.0162 | 0.0073 | 0.0017 | 0.0038 | 0.0121 |
| rs1218822 | 0.0296 | 0.0168 | 0.0076 | 0.0017 | 0.0152 | 0.0123 |
| rs12189178 | -0.0298 | 0.0364 | 0.0201 | 0.0046 | 0.0472 | 0.0328 |
| rs12206564 | 0.006 | 0.0113 | 0.0078 | 0.0017 | 6.00E-04 | 0.0116 |
| rs1227247 | 0.0309 | 0.0102 | 0.0083 | 0.0019 | 0.0192 | 0.0122 |
| rs12299509 | 0.0467 | -0.0032 | 0.0073 | 0.0017 | 0.0189 | 0.0119 |
| rs12299814 | -0.0153 | -0.0157 | 0.0091 | 0.002 | -0.0118 | 0.0122 |
| rs12325419 | -0.0115 | -0.0188 | 0.0121 | 0.0027 | -0.0318 | 0.0186 |
| rs12364470 | 0.0301 | 0.0178 | 0.0096 | 0.0022 | 0.0095 | 0.0139 |
| rs12369179 | 0.0248 | -0.0359 | 0.0136 | 0.0031 | -0.0141 | 0.021 |
| rs12411886 | 0.011 | 0.0271 | 0.0129 | 0.003 | -0.0671 | 0.0208 |
| rs1241986 | -0.0235 | -0.0139 | 0.011 | 0.0024 | -0.0135 | 0.0152 |
| rs12422552 | -0.0358 | -0.0134 | 0.0089 | 0.002 | -0.0068 | 0.0142 |
| rs12429545 | 0.0542 | 0.0316 | 0.0107 | 0.0025 | 0.0211 | 0.0168 |
| rs12439798 | 0.0163 | 0.0125 | 0.0079 | 0.0017 | 0.0171 | 0.0124 |
| rs12443621 | 0.0241 | 0.0096 | 0.0078 | 0.0017 | -0.0205 | 0.0118 |
| rs12446632 | -0.0233 | -0.0352 | 0.0105 | 0.0024 | -0.0126 | 0.0176 |
| rs12448257 | 0.033 | 0.0184 | 0.0089 | 0.002 | 0.0022 | 0.0158 |
| rs12448738 | 0.0179 | 0.0168 | 0.0115 | 0.0025 | 0.0059 | 0.0152 |
| rs12468863 | -0.0149 | -0.0153 | 0.0073 | 0.0016 | 0.0018 | 0.0117 |
| rs12477088 | -0.0177 | -0.0145 | 0.008 | 0.0017 | -0.0039 | 0.0118 |
| rs12488237 | 0.0181 | 0.0235 | 0.0156 | 0.0036 | 0.0655 | 0.0326 |
| rs12502727 | 0.0089 | 0.016 | 0.0126 | 0.0028 | 0.0227 | 0.018 |
| rs12538435 | -0.0183 | -0.0135 | 0.0089 | 0.002 | -0.0095 | 0.0125 |
| rs12564992 | 0.0153 | 0.0196 | 0.0114 | 0.0026 | 0.0528 | 0.0213 |
| rs12593036 | -0.0276 | -0.0154 | 0.0086 | 0.0019 | -0.0092 | 0.013 |
| rs12595158 | -0.0769 | -0.0394 | 0.0284 | 0.0054 | -0.0441 | 0.0192 |
| rs12595749 | -0.0143 | -0.0141 | 0.0079 | 0.0017 | -0.0127 | 0.0118 |
| rs12602912 | 0.0383 | 0.0176 | 0.0089 | 0.0021 | 0.0246 | 0.0136 |
| rs12604935 | 0.0256 | 0.011 | 0.008 | 0.0018 | 0.0285 | 0.0134 |
| rs12615778 | -0.0077 | -0.0104 | 0.0086 | 0.0019 | 0.0073 | 0.0118 |
| rs12617659 | -0.0685 | 0.002 | 0.0103 | 0.0023 | 0.0164 | 0.0164 |
| rs12620249 | 0.0212 | 0.0135 | 0.0109 | 0.0025 | -0.002 | 0.026 |
| rs12629015 | -0.0095 | -0.0135 | 0.0102 | 0.0023 | 0.0097 | 0.0132 |
| rs12630999 | 0.0264 | 0.0175 | 0.0084 | 0.0019 | -0.0032 | 0.0161 |
| rs1263627 | 0.0186 | 0.0139 | 0.0091 | 0.002 | -0.0095 | 0.0133 |
| rs12652212 | 0.0132 | 0.0123 | 0.0073 | 0.0017 | 0.0135 | 0.0121 |
| rs1266874 | 0.0125 | 0.014 | 0.0082 | 0.0018 | -0.0045 | 0.0116 |
| rs12675063 | 0.0145 | 0.0156 | 0.0114 | 0.0026 | -0.0325 | 0.0235 |
| rs12680842 | 0.0082 | -0.0133 | 0.0077 | 0.0018 | -0.0294 | 0.0118 |
| rs12681990 | 0.0634 | -0.0017 | 0.0096 | 0.0022 | 0.0339 | 0.0167 |
| rs12718572 | -0.0252 | -0.0117 | 0.008 | 0.0018 | -0.0081 | 0.0125 |
| rs12759296 | -0.0044 | -0.0104 | 0.0084 | 0.0018 | -0.0128 | 0.0121 |
| rs12762034 | 0.0091 | 0.024 | 0.0146 | 0.0032 | -0.0072 | 0.0197 |
| rs12776880 | -0.0218 | -0.0128 | 0.0086 | 0.0019 | -0.0093 | 0.012 |
| rs1277723 | 0.0098 | 0.0113 | 0.0088 | 0.002 | 0.0057 | 0.0134 |
| rs12779328 | -0.0102 | 0.0105 | 0.0086 | 0.0019 | -0.0014 | 0.014 |
| rs1285997 | -0.0289 | 0.0142 | 0.0088 | 0.0019 | 0.0205 | 0.0132 |
| rs12885454 | -0.0159 | -0.0185 | 0.0074 | 0.0017 | 0.0063 | 0.0121 |
| rs12888545 | 0.015 | 0.0136 | 0.0091 | 0.002 | -0.0161 | 0.0137 |
| rs12888955 | -0.0345 | -0.0178 | 0.0082 | 0.0018 | -0.0346 | 0.012 |
| rs12905439 | 0.0013 | -0.0118 | 0.0082 | 0.0018 | 0.004 | 0.0123 |
| rs12939549 | -0.0177 | -0.018 | 0.0072 | 0.0016 | 0.0156 | 0.0122 |
| rs12945601 | -0.048 | 0.0078 | 0.008 | 0.0018 | 0.0076 | 0.0117 |
| rs12953970 | 0.0171 | 0.0125 | 0.0101 | 0.0023 | 0.0082 | 0.0144 |
| rs1296328 | -0.0412 | -0.0179 | 0.0079 | 0.0018 | 0.0048 | 0.0116 |
| rs12989476 | 0.0035 | 0.013 | 0.0082 | 0.0018 | -0.011 | 0.0134 |
| rs13012099 | -0.0093 | -0.014 | 0.0083 | 0.0018 | 0.0137 | 0.0129 |
| rs13021737 | 0.057 | 0.0574 | 0.0097 | 0.0021 | -0.0164 | 0.0158 |
| rs13034320 | 0.0056 | 0.0114 | 0.009 | 0.0021 | 0.0179 | 0.0135 |
| rs13072095 | -0.0215 | 0.0095 | 0.0077 | 0.0017 | 0.0038 | 0.0132 |
| rs13072412 | -0.0213 | -0.0108 | 0.0079 | 0.0017 | 0.0065 | 0.0117 |
| rs13072731 | -0.0015 | 0.0105 | 0.008 | 0.0018 | 0.0127 | 0.0117 |
| rs13110266 | -0.0208 | -0.0117 | 0.0073 | 0.0017 | -0.0201 | 0.0118 |
| rs13159555 | 0.028 | 0.0119 | 0.0096 | 0.0021 | 0.0074 | 0.0157 |
| rs13168288 | -0.0426 | -0.0133 | 0.0097 | 0.002 | -0.0162 | 0.0127 |
| rs13174863 | 0.0076 | 0.0192 | 0.0102 | 0.0023 | -0.0034 | 0.0163 |
| rs13184896 | -0.0062 | -0.0133 | 0.0073 | 0.0016 | 0.001 | 0.0117 |
| rs13191362 | -0.0276 | -0.0236 | 0.0112 | 0.0025 | -0.0217 | 0.0242 |
| rs13201877 | 0.0096 | 0.0152 | 0.0105 | 0.0024 | 0.0162 | 0.0158 |
| rs1320903 | 0.0411 | 0.0216 | 0.0084 | 0.0018 | -0.001 | 0.0128 |
| rs1321432 | 0.0022 | 0.0201 | 0.0082 | 0.0018 | -0.0098 | 0.0124 |
| rs13227658 | -0.0161 | -0.0157 | 0.0079 | 0.0017 | -0.0238 | 0.0116 |
| rs13239186 | 0.0539 | 0.0035 | 0.0085 | 0.0019 | 0.0036 | 0.0121 |
| rs13240600 | -0.0133 | -0.0204 | 0.0109 | 0.0024 | 0.0229 | 0.0139 |
| rs13250058 | 0.0096 | 0.0112 | 0.0078 | 0.0018 | 0.0151 | 0.0125 |
| rs13263601 | 0.0279 | 0.0154 | 0.0082 | 0.0018 | 0.011 | 0.0132 |
| rs13267015 | 0.0102 | 0.0105 | 0.0087 | 0.0019 | 0.0098 | 0.0139 |
| rs1327259 | -0.0234 | -0.0155 | 0.0081 | 0.0018 | -0.0021 | 0.0117 |
| rs13290794 | -0.0293 | -0.0141 | 0.0081 | 0.0018 | -0.0295 | 0.0129 |
| rs13292976 | 0.0197 | 0.0131 | 0.0078 | 0.0017 | 0.0135 | 0.0117 |
| rs13329567 | -0.0283 | -0.0293 | 0.0086 | 0.002 | -0.0166 | 0.0158 |
| rs1333039 | 0.0534 | -0.0039 | 0.0074 | 0.0017 | -0.0099 | 0.0119 |
| rs1336486 | 0.0071 | 0.0141 | 0.0084 | 0.0018 | -0.0045 | 0.0118 |
| rs13389219 | -0.0722 | 0.0112 | 0.0074 | 0.0017 | -0.0147 | 0.0122 |
| rs13417156 | 0.0022 | 0.0144 | 0.0078 | 0.0017 | -0.0152 | 0.0118 |
| rs13432055 | 0.03 | 0.0117 | 0.0078 | 0.0018 | 0.0053 | 0.0137 |
| rs1359790 | -0.0796 | -0.0013 | 0.008 | 0.0018 | -0.0013 | 0.0128 |
| rs1363695 | -0.0185 | -0.013 | 0.0097 | 0.0021 | 0.0052 | 0.013 |
| rs1365466 | -0.0237 | -0.0137 | 0.0082 | 0.0019 | -0.0057 | 0.0147 |
| rs1371108 | 0.007 | 0.0119 | 0.0084 | 0.0018 | 0.0116 | 0.0127 |
| rs1394 | -0.034 | -0.0154 | 0.0079 | 0.0017 | 0.0048 | 0.0158 |
| rs1394879 | 0.0146 | 0.0101 | 0.0078 | 0.0017 | 0.0069 | 0.0118 |
| rs1399471 | 0.0158 | 0.0131 | 0.009 | 0.002 | 0.0154 | 0.0134 |
| rs1402025 | 0.014 | 0.0121 | 0.0093 | 0.0021 | 0.0082 | 0.0135 |
| rs1409818 | 0.0281 | 0.0201 | 0.013 | 0.0029 | -0.0171 | 0.0235 |
| rs1412235 | 0.0299 | 0.0246 | 0.0076 | 0.0017 | -0.0018 | 0.012 |
| rs1421334 | -0.0363 | -0.0125 | 0.0079 | 0.0018 | 0.007 | 0.0119 |
| rs14291 | 0.0058 | 0.0105 | 0.0081 | 0.0018 | 0.011 | 0.0118 |
| rs1430387 | -0.0198 | -0.0114 | 0.0079 | 0.0017 | 0.0156 | 0.0116 |
| rs1431659 | -0.0096 | -0.0196 | 0.0088 | 0.0019 | -0.0081 | 0.0135 |
| rs1436344 | 0.0112 | 0.0141 | 0.008 | 0.0017 | 0.012 | 0.012 |
| rs1452075 | 0.0333 | 0.0141 | 0.0081 | 0.0018 | -0.0017 | 0.0128 |
| rs1454148 | 0.0087 | 0.0106 | 0.0087 | 0.0019 | -0.0027 | 0.0139 |
| rs1465900 | -0.0379 | -0.0125 | 0.0089 | 0.002 | -0.0129 | 0.0143 |
| rs1476322 | 0.02 | 0.0101 | 0.0079 | 0.0017 | -0.0036 | 0.0116 |
| rs1477199 | 0.0231 | 0.0228 | 0.0104 | 0.0024 | 0.0034 | 0.0183 |
| rs1485038 | 0.0111 | 0.0143 | 0.01 | 0.0022 | -0.0228 | 0.0144 |
| rs1492767 | 0.0092 | 0.0094 | 0.0072 | 0.0016 | 0.0054 | 0.0117 |
| rs1496653 | -0.0769 | 8.00E-04 | 0.0088 | 0.002 | 0.0054 | 0.0125 |
| rs1503526 | 0.0191 | 0.014 | 0.0078 | 0.0017 | 0.0092 | 0.0118 |
| rs1511471 | 0.0152 | 0.0113 | 0.0083 | 0.0019 | 0.0121 | 0.0123 |
| rs1512914 | -0.0049 | -0.0126 | 0.008 | 0.0018 | -4.00E-04 | 0.012 |
| rs1522569 | -0.0123 | -0.0164 | 0.0102 | 0.0022 | -0.0313 | 0.0211 |
| rs1523768 | -0.0234 | -0.011 | 0.0076 | 0.0017 | 0.0011 | 0.0126 |
| rs1524277 | -0.0087 | -0.0106 | 0.0079 | 0.0018 | -0.0078 | 0.0117 |
| rs1528435 | 0.0318 | 0.0164 | 0.0075 | 0.0017 | 0.0239 | 0.0121 |
| rs1530344 | 0.0024 | 0.01 | 0.0078 | 0.0017 | -0.0085 | 0.0116 |
| rs1530559 | 0.0144 | -0.0097 | 0.0075 | 0.0017 | -0.0106 | 0.0117 |
| rs1552224 | -0.1034 | 0.0111 | 0.0101 | 0.0025 | 0.0265 | 0.0136 |
| rs1554622 | 0.0053 | 0.0114 | 0.0072 | 0.0016 | 0.0354 | 0.0116 |
| rs155510 | 0.019 | 0.0165 | 0.0098 | 0.0021 | -0.0081 | 0.0139 |
| rs1579200 | -0.003 | -0.0136 | 0.0103 | 0.0023 | -0.0275 | 0.0138 |
| rs1579557 | 0.0371 | 0.0213 | 0.0084 | 0.0019 | -0.013 | 0.0123 |
| rs159032 | 0.0142 | 0.0129 | 0.009 | 0.002 | 0.0114 | 0.0148 |
| rs1625427 | -0.0161 | -0.013 | 0.0082 | 0.0018 | 0.0152 | 0.0132 |
| rs1657930 | 0.0114 | -0.0123 | 0.0099 | 0.0022 | 0.0196 | 0.0161 |
| rs1658820 | 0.0017 | 0.0141 | 0.0091 | 0.0021 | -0.002 | 0.013 |
| rs1668633 | -4.00E-04 | 0.01 | 0.0079 | 0.0017 | -0.0135 | 0.0117 |
| rs16851483 | 0.0369 | 0.0369 | 0.0157 | 0.0035 | 0.0063 | 0.0271 |
| rs16864515 | -0.0086 | 0.0186 | 0.013 | 0.0029 | -0.0185 | 0.021 |
| rs16871902 | 0.0112 | 0.0125 | 0.0078 | 0.0017 | 0.0035 | 0.0116 |
| rs16903232 | 0.0096 | 0.0167 | 0.0122 | 0.0028 | -0.0178 | 0.0161 |
| rs16903285 | 0.0434 | 0.0331 | 0.0114 | 0.0026 | -0.003 | 0.0188 |
| rs16906845 | -0.0276 | -0.0225 | 0.0164 | 0.0038 | -0.0017 | 0.0211 |
| rs16907751 | -0.0248 | -0.0209 | 0.0126 | 0.003 | 0.0192 | 0.018 |
| rs16932761 | -0.0288 | -0.014 | 0.0091 | 0.002 | -0.0058 | 0.0126 |
| rs16940823 | -0.0192 | -0.0146 | 0.0099 | 0.0023 | 0.0068 | 0.0151 |
| rs16958372 | 0.0212 | 0.0141 | 0.0107 | 0.0025 | 0.0459 | 0.0152 |
| rs16965225 | 0.0482 | 0.0216 | 0.0148 | 0.0034 | -0.004 | 0.0293 |
| rs16966801 | 0.0102 | 0.0156 | 0.0104 | 0.0022 | 0.0194 | 0.0187 |
| rs16988333 | -0.0745 | 0.0047 | 0.013 | 0.0029 | -0.0235 | 0.0208 |
| rs17001561 | 0.0129 | 0.0151 | 0.0101 | 0.0023 | -0.0085 | 0.0182 |
| rs17014375 | 0.0229 | 0.0172 | 0.0114 | 0.0025 | 0.0158 | 0.019 |
| rs17015701 | 0.0322 | 0.0135 | 0.0091 | 0.0021 | -0.0389 | 0.0191 |
| rs1704190 | 0.0149 | 0.0098 | 0.0081 | 0.0018 | -0.0181 | 0.0119 |
| rs17056301 | -0.0041 | 0.0118 | 0.009 | 0.002 | 0.0176 | 0.014 |
| rs17069831 | 0.008 | -0.0109 | 0.0087 | 0.0019 | -0.0073 | 0.0129 |
| rs17094222 | 0.0196 | 0.0181 | 0.0087 | 0.002 | 0.0133 | 0.0137 |
| rs17096510 | -0.0023 | -0.0148 | 0.0115 | 0.0026 | -0.0481 | 0.0208 |
| rs17105272 | -0.0135 | 0.011 | 0.0084 | 0.0019 | -0.0261 | 0.0124 |
| rs17119937 | 0.001 | 0.0212 | 0.0151 | 0.0036 | -4.00E-04 | 0.0259 |
| rs17120367 | 0.0172 | 0.0225 | 0.0169 | 0.0037 | -0.0122 | 0.022 |
| rs17168486 | 0.0742 | 0.0014 | 0.0094 | 0.0022 | -0.0048 | 0.0145 |
| rs17171818 | 0.0169 | 0.0117 | 0.0096 | 0.0021 | -0.0128 | 0.0137 |
| rs17200912 | 0.0137 | -0.0145 | 0.0093 | 0.002 | 0.0065 | 0.0134 |
| rs17201143 | 0.0068 | -0.0103 | 0.0083 | 0.0019 | -0.0229 | 0.0128 |
| rs17203016 | -0.009 | 0.015 | 0.0091 | 0.002 | 0.0401 | 0.0145 |
| rs17236194 | 0.0084 | 0.0153 | 0.0102 | 0.0024 | 0.0164 | 0.0141 |
| rs17272434 | -0.0052 | -0.0105 | 0.0077 | 0.0018 | 0.0073 | 0.0125 |
| rs17334919 | -0.1398 | 0.0026 | 0.0128 | 0.0028 | -0.0233 | 0.0274 |
| rs17391694 | -0.0122 | 0.0317 | 0.0109 | 0.0025 | 0.0243 | 0.0173 |
| rs17399237 | -0.0154 | -0.0129 | 0.0079 | 0.0017 | 0.0015 | 0.0119 |
| rs17405819 | -0.0177 | -0.0215 | 0.0079 | 0.0018 | 0.0123 | 0.0128 |
| rs17411031 | -0.045 | -0.0044 | 0.0081 | 0.0018 | -0.0062 | 0.0135 |
| rs17424278 | 0.0308 | 0.017 | 0.0127 | 0.0028 | 0.015 | 0.0218 |
| rs17446091 | 0.0092 | 0.0123 | 0.0086 | 0.002 | -0.0288 | 0.0166 |
| rs17450772 | 0.0257 | 0.0189 | 0.0125 | 0.003 | -0.0211 | 0.0167 |
| rs17513613 | 0.0268 | 0.0186 | 0.0077 | 0.0018 | -0.0058 | 0.0122 |
| rs17522122 | 0.0403 | 0.0159 | 0.0074 | 0.0017 | -0.0097 | 0.0118 |
| rs17531363 | 0.0038 | -0.0133 | 0.0085 | 0.0019 | 0.0034 | 0.0117 |
| rs17599948 | -0.0022 | -0.013 | 0.0098 | 0.0022 | 0.0094 | 0.0153 |
| rs17619973 | -0.0191 | -0.02 | 0.014 | 0.0032 | 0.0171 | 0.029 |
| rs17636031 | -0.0071 | 0.016 | 0.0082 | 0.0019 | -0.043 | 0.0163 |
| rs17681708 | 0.0389 | 0.0106 | 0.0083 | 0.0018 | 0.0107 | 0.0124 |
| rs17695092 | 0.0137 | -0.0106 | 0.008 | 0.0018 | 0.0101 | 0.0118 |
| rs17709991 | 0.0023 | 0.011 | 0.0081 | 0.0018 | -0.0098 | 0.013 |
| rs17720922 | 0.0148 | 0.0131 | 0.0099 | 0.0022 | -0.0056 | 0.0134 |
| rs17757975 | -0.0088 | -0.0143 | 0.0111 | 0.0024 | -0.0234 | 0.0186 |
| rs17767510 | 0.0228 | 0.0129 | 0.0102 | 0.0023 | 0.0045 | 0.0161 |
| rs17789218 | 0.0027 | 0.013 | 0.0083 | 0.0019 | -3.00E-04 | 0.0143 |
| rs17791483 | -0.102 | -0.0064 | 0.0147 | 0.0033 | -0.0136 | 0.0177 |
| rs17805532 | 0.0392 | 0.0226 | 0.0167 | 0.0039 | 0.0106 | 0.0232 |
| rs17806379 | -0.0354 | -0.0258 | 0.0103 | 0.0022 | -0.0193 | 0.016 |
| rs17820822 | -0.0117 | -0.0143 | 0.0082 | 0.0018 | -0.0089 | 0.0127 |
| rs1784460 | 0.0111 | 0.0132 | 0.0079 | 0.0018 | -0.0035 | 0.0128 |
| rs1787267 | -0.0602 | -0.0237 | 0.0166 | 0.0036 | 0.0129 | 0.0241 |
| rs1801214 | 0.0903 | 0.0044 | 0.0074 | 0.0017 | 0.0159 | 0.0118 |
| rs1805207 | 0.0144 | 0.011 | 0.0084 | 0.0018 | 0.0115 | 0.0125 |
| rs1819844 | -0.0397 | -0.0143 | 0.0092 | 0.0021 | -0.0159 | 0.013 |
| rs1829130 | 0.0054 | 0.011 | 0.0085 | 0.0019 | 0.0074 | 0.0121 |
| rs1852006 | -0.0044 | -0.0156 | 0.0082 | 0.0018 | 0.0026 | 0.012 |
| rs1863652 | -0.0155 | -0.0115 | 0.008 | 0.0018 | -0.0041 | 0.0127 |
| rs1865341 | 0.0058 | 0.0128 | 0.0093 | 0.002 | -0.0119 | 0.0127 |
| rs1865989 | 0.0129 | 0.01 | 0.0079 | 0.0018 | -0.0018 | 0.012 |
| rs1884389 | -0.0106 | -0.0103 | 0.0079 | 0.0017 | 0.0053 | 0.0117 |
| rs1885728 | -0.0017 | 0.0108 | 0.0085 | 0.0019 | -0.0306 | 0.0125 |
| rs1891215 | 0.003 | 0.0117 | 0.0078 | 0.0017 | -0.0119 | 0.0116 |
| rs1895957 | 0.0218 | 0.0171 | 0.0095 | 0.0021 | 0.0272 | 0.0138 |
| rs1899689 | 0.0217 | 0.0117 | 0.0073 | 0.0016 | 0.0167 | 0.0118 |
| rs1899898 | -0.0171 | -0.0113 | 0.0083 | 0.0018 | -0.0058 | 0.012 |
| rs1899951 | -0.1118 | 0.017 | 0.0109 | 0.0024 | 7.00E-04 | 0.0155 |
| rs1912631 | -0.0021 | -0.0119 | 0.0079 | 0.0018 | 0.0056 | 0.0121 |
| rs1927790 | 0.0138 | 0.0148 | 0.0073 | 0.0016 | 0.0082 | 0.0116 |
| rs1928295 | -0.0267 | -0.0141 | 0.0072 | 0.0016 | -0.0036 | 0.0116 |
| rs1937683 | 0.0071 | 0.0109 | 0.0086 | 0.0018 | -5.00E-04 | 0.0123 |
| rs1941697 | 0.0214 | 0.0123 | 0.0079 | 0.0017 | 0.021 | 0.0116 |
| rs1945941 | -0.0026 | -0.0114 | 0.0078 | 0.0018 | -0.0041 | 0.0123 |
| rs1964927 | 0.0122 | 0.0124 | 0.0082 | 0.0018 | 0.0108 | 0.0119 |
| rs1982441 | -0.015 | 0.0175 | 0.0116 | 0.0026 | -0.0164 | 0.015 |
| rs1990573 | 0.0322 | 0.013 | 0.0078 | 0.0018 | 0.0076 | 0.0124 |
| rs1993709 | 0.0378 | 0.0331 | 0.0096 | 0.0021 | 0.0253 | 0.0168 |
| rs2000 | -0.0091 | -0.0281 | 0.0225 | 0.0051 | 0.0747 | 0.0534 |
| rs2007231 | -0.0159 | -0.0104 | 0.0081 | 0.0018 | 0.0071 | 0.0126 |
| rs200810 | -0.0184 | -0.0136 | 0.0074 | 0.0017 | -0.002 | 0.0119 |
| rs2009416 | -0.0109 | -0.0121 | 0.0081 | 0.0018 | 0.0026 | 0.0117 |
| rs2027524 | -0.0153 | -0.0175 | 0.0133 | 0.003 | 0.0377 | 0.0236 |
| rs2029085 | -0.0185 | -0.0164 | 0.0126 | 0.0028 | -0.0133 | 0.0161 |
| rs2035831 | -0.0218 | -0.0108 | 0.0083 | 0.0018 | -0.0156 | 0.0119 |
| rs2044469 | 0.0084 | 0.0144 | 0.0082 | 0.0018 | -0.0023 | 0.0125 |
| rs2051559 | 0.0375 | 0.0176 | 0.0114 | 0.0026 | 0.0206 | 0.0187 |
| rs2053865 | 0.0208 | 0.01 | 0.0079 | 0.0017 | -0.0053 | 0.0118 |
| rs2057316 | -0.0069 | -0.0229 | 0.0187 | 0.0042 | 0.006 | 0.0273 |
| rs2061813 | -0.0107 | -0.0112 | 0.0091 | 0.002 | -0.009 | 0.0142 |
| rs2063177 | 0.0166 | 0.012 | 0.0082 | 0.0018 | -0.0134 | 0.0121 |
| rs2065418 | -0.0298 | -0.0166 | 0.0081 | 0.0018 | -0.0073 | 0.0126 |
| rs2077460 | -0.0097 | -0.0114 | 0.008 | 0.0018 | -0.0185 | 0.0117 |
| rs208015 | 0.0308 | -0.0356 | 0.0154 | 0.0034 | 0.0395 | 0.0185 |
| rs2080454 | -0.0248 | -0.0129 | 0.0074 | 0.0017 | 0.0061 | 0.0116 |
| rs2094474 | 0.0206 | 0.0114 | 0.0087 | 0.0019 | -0.0042 | 0.0128 |
| rs2119753 | -0.013 | -0.0102 | 0.0081 | 0.0018 | -0.0087 | 0.0119 |
| rs2122042 | 0.0287 | 0.0235 | 0.0091 | 0.002 | -0.0125 | 0.0154 |
| rs2143253 | -0.0225 | -0.0188 | 0.0119 | 0.0026 | -0.0322 | 0.0194 |
| rs2143624 | 0.0059 | 0.0098 | 0.0075 | 0.0017 | 0.013 | 0.0118 |
| rs2155645 | -0.025 | -0.0116 | 0.0082 | 0.0019 | -0.0199 | 0.0127 |
| rs215634 | -0.0256 | -0.0152 | 0.008 | 0.0018 | -0.0237 | 0.013 |
| rs2160077 | 0.0087 | 0.0093 | 0.0072 | 0.0016 | -0.006 | 0.0119 |
| rs2161097 | 0.0208 | 0.0096 | 0.008 | 0.0017 | 0.0067 | 0.0119 |
| rs2162524 | 0.0175 | 0.0155 | 0.0083 | 0.0018 | -0.0021 | 0.0126 |
| rs2170382 | 0.0321 | 0.0172 | 0.0122 | 0.0027 | 0.0342 | 0.0183 |
| rs217433 | -0.0087 | 0.0117 | 0.0091 | 0.0021 | 0.0257 | 0.0147 |
| rs2174367 | -0.0035 | -0.0121 | 0.0085 | 0.0019 | 0.0247 | 0.0126 |
| rs217671 | 0.0197 | 0.0144 | 0.0088 | 0.0019 | 0.028 | 0.0142 |
| rs2185027 | 0.0344 | 0.0135 | 0.0079 | 0.0018 | -0.0037 | 0.0123 |
| rs2190788 | 0.0165 | 0.0141 | 0.0083 | 0.0019 | 0.0247 | 0.0125 |
| rs2190890 | -0.0299 | -0.0111 | 0.009 | 0.002 | -0.0135 | 0.0124 |
| rs2191348 | 0.0652 | -0.0018 | 0.0073 | 0.0016 | 0.0076 | 0.0117 |
| rs2198679 | -0.0247 | -0.0107 | 0.0079 | 0.0017 | 7.00E-04 | 0.0124 |
| rs2228213 | -0.0051 | -0.0139 | 0.0075 | 0.0017 | 0.0088 | 0.0122 |
| rs223051 | 0.013 | 0.0112 | 0.0083 | 0.0018 | -0.0177 | 0.0128 |
| rs2235564 | 0.0316 | 0.0131 | 0.0082 | 0.0018 | -0.0066 | 0.0122 |
| rs2246012 | 0.0527 | 0.0158 | 0.0094 | 0.0022 | 0.0082 | 0.0138 |
| rs2261181 | 0.0985 | -0.0011 | 0.0118 | 0.0028 | 0.0033 | 0.0224 |
| rs2269828 | -0.0206 | -0.0105 | 0.0078 | 0.0018 | 0.0034 | 0.0125 |
| rs2270778 | 0.0214 | -0.0097 | 0.0074 | 0.0017 | -0.0184 | 0.0118 |
| rs2274550 | -0.0218 | -0.014 | 0.0114 | 0.0025 | -0.0081 | 0.0162 |
| rs2281819 | -0.0098 | -0.016 | 0.0093 | 0.002 | 0.0241 | 0.0127 |
| rs2282231 | 0.054 | 0.0165 | 0.0095 | 0.0021 | 0.0199 | 0.0148 |
| rs2283093 | -3.00E-04 | 0.0127 | 0.0097 | 0.0021 | 0.0095 | 0.0148 |
| rs2289379 | -0.0121 | -0.0135 | 0.008 | 0.0018 | -0.0243 | 0.0117 |
| rs2291487 | 0.0048 | 0.0126 | 0.0097 | 0.0022 | -0.0544 | 0.0178 |
| rs2292662 | -0.0629 | -0.0041 | 0.0111 | 0.0024 | -0.0365 | 0.0141 |
| rs2294120 | -0.0443 | -3.00E-04 | 0.0079 | 0.0017 | -0.0159 | 0.0117 |
| rs2303083 | -0.0165 | -0.0145 | 0.01 | 0.0022 | -0.0129 | 0.017 |
| rs2306537 | 0.0252 | 0.0133 | 0.0084 | 0.0019 | 0.0108 | 0.0133 |
| rs2307022 | -0.0049 | -0.0135 | 0.0077 | 0.0017 | -0.0141 | 0.0124 |
| rs2307111 | -0.0407 | -0.0265 | 0.0074 | 0.0016 | -0.0036 | 0.0117 |
| rs2317299 | -0.0214 | -0.0106 | 0.0079 | 0.0017 | -0.0185 | 0.0117 |
| rs2322622 | 0.0228 | 0.0094 | 0.0074 | 0.0017 | 0.0223 | 0.0117 |
| rs2357760 | 0.0168 | 0.0145 | 0.0077 | 0.0017 | 0.0082 | 0.0123 |
| rs236527 | -0.0142 | -0.0107 | 0.0086 | 0.0019 | -0.0313 | 0.013 |
| rs2365389 | -0.0099 | -0.0174 | 0.0073 | 0.0017 | -6.00E-04 | 0.0117 |
| rs2367112 | -0.0054 | -0.0119 | 0.0072 | 0.0016 | -0.0147 | 0.0116 |
| rs2371984 | 0.0035 | -0.0144 | 0.0117 | 0.0026 | 0.0404 | 0.0182 |
| rs2421016 | -0.0458 | 0.0042 | 0.0071 | 0.0016 | 0.0135 | 0.012 |
| rs2425024 | 0.0068 | 0.0103 | 0.0084 | 0.0018 | 5.00E-04 | 0.0124 |
| rs2425840 | 0.0142 | 0.0119 | 0.008 | 0.0018 | 0.0165 | 0.0123 |
| rs2429150 | 0.0152 | 0.0111 | 0.008 | 0.0018 | -0.0122 | 0.0117 |
| rs243019 | 0.0566 | 0.0016 | 0.0071 | 0.0016 | -0.0138 | 0.0117 |
| rs2440452 | -0.0019 | -0.0142 | 0.0115 | 0.0026 | 0.0267 | 0.0188 |
| rs2467594 | 1.00E-04 | 0.0122 | 0.0081 | 0.0018 | -0.0063 | 0.0119 |
| rs2479958 | -0.0239 | -0.0154 | 0.008 | 0.0018 | -0.0012 | 0.0117 |
| rs248139 | -0.0108 | 0.0133 | 0.0097 | 0.0022 | -0.0187 | 0.0144 |
| rs2481665 | -0.0221 | -0.0161 | 0.0075 | 0.0016 | -0.0233 | 0.0121 |
| rs2543132 | 0.0064 | 0.0146 | 0.0101 | 0.0022 | -6.00E-04 | 0.0159 |
| rs2605603 | -0.019 | -0.0103 | 0.0072 | 0.0016 | -0.0107 | 0.0116 |
| rs2612579 | 0.0138 | 0.0108 | 0.0085 | 0.0019 | -0.0016 | 0.0131 |
| rs2616132 | 0.0455 | 6.00E-04 | 0.0078 | 0.0018 | 0.0158 | 0.0118 |
| rs2616192 | 0.0165 | 0.0125 | 0.008 | 0.0019 | 0.0149 | 0.0119 |
| rs262130 | 4.00E-04 | 0.0127 | 0.01 | 0.0023 | -0.0089 | 0.0144 |
| rs263041 | -0.0226 | -0.0124 | 0.0081 | 0.0018 | 0.0162 | 0.0117 |
| rs2631681 | -0.0188 | -0.0113 | 0.0078 | 0.0017 | 0.0181 | 0.0126 |
| rs2633310 | -0.0443 | -0.0046 | 0.0079 | 0.0017 | 0.0199 | 0.0117 |
| rs2694047 | 0.0156 | 0.0188 | 0.0092 | 0.002 | -1.00E-04 | 0.0128 |
| rs2710323 | 0.0058 | 0.0141 | 0.0072 | 0.0016 | -0.0115 | 0.0116 |
| rs2717926 | -0.0053 | 0.0097 | 0.0077 | 0.0017 | -0.0191 | 0.0128 |
| rs2733287 | 0.0172 | 0.0157 | 0.0078 | 0.0017 | -0.0059 | 0.0116 |
| rs273697 | 0.0059 | -0.0098 | 0.0079 | 0.0017 | -0.006 | 0.0116 |
| rs2744974 | -0.0062 | 0.0249 | 0.0076 | 0.0018 | 0.0457 | 0.0124 |
| rs2796441 | -0.0715 | 0.0066 | 0.0073 | 0.0017 | 0.012 | 0.0117 |
| rs2820311 | 0.0294 | 0.0235 | 0.0083 | 0.0018 | -0.0021 | 0.0127 |
| rs2820426 | 0.0521 | -0.0081 | 0.0073 | 0.0017 | 0.0222 | 0.0119 |
| rs2832283 | 0.0041 | 0.0115 | 0.0086 | 0.002 | -0.0038 | 0.0133 |
| rs2836964 | -0.011 | -0.011 | 0.0082 | 0.0018 | -0.0326 | 0.0138 |
| rs2837398 | -0.003 | 0.0114 | 0.0079 | 0.0018 | -0.012 | 0.0123 |
| rs284227 | -0.005 | -0.0147 | 0.0083 | 0.0019 | 3.00E-04 | 0.0123 |
| rs2850969 | 0.0277 | 0.0165 | 0.0111 | 0.0024 | 0.0198 | 0.0157 |
| rs2865805 | 0.0095 | 0.0111 | 0.0085 | 0.0019 | -0.0027 | 0.0125 |
| rs2875762 | 0.0265 | 0.0139 | 0.009 | 0.002 | 0.0438 | 0.0186 |
| rs2876248 | -0.0159 | -0.0105 | 0.0082 | 0.0018 | 0.0195 | 0.0125 |
| rs2890652 | 0.0236 | 0.017 | 0.0104 | 0.0023 | 0.023 | 0.0135 |
| rs2899663 | -3.00E-04 | 0.0092 | 0.0071 | 0.0016 | -0.0127 | 0.0117 |
| rs2907948 | -0.0232 | -0.0141 | 0.0085 | 0.0019 | -0.0099 | 0.0156 |
| rs2925979 | -0.0534 | 4.00E-04 | 0.0078 | 0.0018 | -0.0114 | 0.0124 |
| rs294704 | -0.0159 | -0.0113 | 0.0088 | 0.0019 | -0.0069 | 0.0137 |
| rs2959592 | -0.0184 | -0.0118 | 0.0092 | 0.0021 | 0.0039 | 0.0137 |
| rs2972144 | 0.0913 | -0.0057 | 0.0075 | 0.0017 | 0.0067 | 0.012 |
| rs2973564 | -0.0241 | -0.0111 | 0.0086 | 0.0019 | 0.0013 | 0.0134 |
| rs298563 | -0.0076 | -0.0127 | 0.0104 | 0.0023 | -0.0038 | 0.0182 |
| rs29941 | 0.0248 | 0.0152 | 0.0077 | 0.0018 | 0.0268 | 0.0119 |
| rs3007105 | 0.0129 | 0.0142 | 0.0072 | 0.0017 | 0.0162 | 0.0122 |
| rs302864 | 0.071 | 0.0064 | 0.0127 | 0.003 | -0.0235 | 0.0235 |
| rs3209570 | -0.0017 | -0.0143 | 0.0082 | 0.0018 | -0.0025 | 0.0119 |
| rs323742 | 0.0787 | 0.041 | 0.0246 | 0.0056 | -0.0547 | 0.0356 |
| rs326896 | -0.0146 | -0.0128 | 0.008 | 0.0018 | -9.00E-04 | 0.0124 |
| rs328049 | -0.0025 | 0.0101 | 0.0079 | 0.0017 | -0.0064 | 0.0117 |
| rs329122 | 0.0372 | -0.0125 | 0.0072 | 0.0017 | -0.0048 | 0.0118 |
| rs329277 | -0.0044 | 0.0099 | 0.0078 | 0.0017 | 0.0082 | 0.0116 |
| rs331949 | 0.0178 | 0.011 | 0.0075 | 0.0017 | 0.0029 | 0.0127 |
| rs33485 | -0.0158 | -0.0158 | 0.0088 | 0.002 | 0.0022 | 0.0135 |
| rs340025 | 0.0204 | 0.0124 | 0.0074 | 0.0017 | -0.0273 | 0.0124 |
| rs340874 | 0.0626 | 0.0019 | 0.0073 | 0.0016 | -0.0124 | 0.0118 |
| rs349088 | -0.0205 | -0.0128 | 0.0078 | 0.0017 | 0.0055 | 0.012 |
| rs3731695 | 0.0094 | 0.0116 | 0.0072 | 0.0016 | -8.00E-04 | 0.0117 |
| rs3736485 | -0.0332 | -0.0134 | 0.0072 | 0.0016 | 0.0102 | 0.0118 |
| rs3754963 | -0.0127 | -0.0123 | 0.0088 | 0.002 | -0.0127 | 0.0126 |
| rs3764835 | -0.0091 | -0.0141 | 0.0105 | 0.0024 | 0.0039 | 0.015 |
| rs3766430 | 0.0131 | 0.0113 | 0.0072 | 0.0016 | -0.0027 | 0.0119 |
| rs3772882 | 0.0219 | 0.0127 | 0.008 | 0.0018 | -0.001 | 0.0117 |
| rs3781099 | 0.0136 | 0.021 | 0.0141 | 0.0031 | 0.0176 | 0.0186 |
| rs3800229 | 0.0153 | 0.0175 | 0.0079 | 0.0018 | 0.016 | 0.0121 |
| rs3802177 | -0.1217 | 0.006 | 0.008 | 0.0018 | -0.0048 | 0.012 |
| rs380857 | -0.0158 | -0.0151 | 0.0121 | 0.0027 | -0.0263 | 0.0151 |
| rs3810291 | 0.0328 | 0.0274 | 0.0087 | 0.0018 | -0.0111 | 0.012 |
| rs3813680 | -0.0064 | -0.0144 | 0.0107 | 0.0024 | -0.0014 | 0.0155 |
| rs3814883 | 0.0295 | 0.0232 | 0.0079 | 0.0017 | 0.0199 | 0.0118 |
| rs3826705 | 0.0018 | 0.0157 | 0.0122 | 0.0027 | -0.0082 | 0.0221 |
| rs3844598 | 0.0015 | 0.0095 | 0.0079 | 0.0017 | -0.0032 | 0.0117 |
| rs3852012 | -0.0099 | -0.0131 | 0.0078 | 0.0018 | -0.0023 | 0.0118 |
| rs3887080 | 0.0276 | 0.0181 | 0.0118 | 0.0026 | 0.0338 | 0.0198 |
| rs3887925 | 0.0474 | -0.0037 | 0.0079 | 0.0017 | 0.0075 | 0.0117 |
| rs3902951 | 0.0258 | 0.0134 | 0.0084 | 0.002 | 0.0313 | 0.0123 |
| rs391300 | 0.0018 | -0.0119 | 0.0075 | 0.0017 | 0.0228 | 0.012 |
| rs3930349 | -0.0153 | -0.0144 | 0.0095 | 0.0021 | -0.0203 | 0.0134 |
| rs40067 | -0.0089 | -0.0266 | 0.0105 | 0.0023 | -0.0084 | 0.0143 |
| rs4012234 | 0.0328 | 0.0141 | 0.0081 | 0.0018 | -0.0085 | 0.0122 |
| rs40245 | -0.0157 | -0.011 | 0.0083 | 0.0018 | -0.0167 | 0.0121 |
| rs4077093 | -0.0226 | -0.0128 | 0.0095 | 0.0022 | -0.0212 | 0.014 |
| rs4082793 | -0.0153 | -0.0122 | 0.008 | 0.0018 | 0.0023 | 0.012 |
| rs4148155 | -0.0056 | -0.0188 | 0.0117 | 0.0026 | 0.0094 | 0.0222 |
| rs4148233 | 0.0052 | 0.0101 | 0.0075 | 0.0017 | 0.0164 | 0.0118 |
| rs4237643 | -0.0377 | -0.0223 | 0.0085 | 0.0019 | 0.0052 | 0.0126 |
| rs427943 | 0.0298 | 0.017 | 0.008 | 0.0017 | 0.0212 | 0.0118 |
| rs429343 | -0.0256 | -0.015 | 0.0081 | 0.0017 | 0.0024 | 0.0117 |
| rs4307239 | 0.0034 | 0.0115 | 0.008 | 0.0017 | -0.0197 | 0.0117 |
| rs4372836 | -0.0022 | -0.0142 | 0.0077 | 0.0018 | -0.0067 | 0.0127 |
| rs4430672 | -0.0061 | -0.0127 | 0.0098 | 0.0022 | 0.023 | 0.0143 |
| rs4432271 | 0.0297 | 0.0228 | 0.0118 | 0.0026 | 0.0171 | 0.0144 |
| rs4472028 | -0.0453 | -0.0045 | 0.0071 | 0.0016 | -0.0093 | 0.0118 |
| rs4482463 | -0.0362 | -0.0331 | 0.0146 | 0.0033 | 0.0153 | 0.0245 |
| rs4518345 | -0.0271 | -0.0117 | 0.0086 | 0.0019 | -0.0055 | 0.0136 |
| rs4521182 | -0.0168 | -0.0104 | 0.0085 | 0.0019 | 0.0134 | 0.0132 |
| rs4523610 | 0.0282 | 0.0123 | 0.0092 | 0.002 | 0.0139 | 0.0134 |
| rs4550409 | -0.0418 | -0.0137 | 0.0112 | 0.0025 | 0.0062 | 0.0236 |
| rs455527 | 3.00E-04 | -0.0199 | 0.015 | 0.0035 | 0.0034 | 0.016 |
| rs4556997 | 0.0161 | 0.0197 | 0.0103 | 0.0024 | -0.0097 | 0.0169 |
| rs459193 | 0.0711 | -0.0015 | 0.0083 | 0.0019 | 0.0312 | 0.0125 |
| rs459552 | -0.0212 | -0.0126 | 0.0085 | 0.002 | -0.0191 | 0.0127 |
| rs460799 | 0.0077 | 0.0118 | 0.0088 | 0.0019 | -0.008 | 0.0126 |
| rs4639527 | 0.0197 | 0.0172 | 0.0084 | 0.0019 | 0.0015 | 0.012 |
| rs4670627 | 0.032 | 0.0105 | 0.0072 | 0.0017 | -0.0231 | 0.0117 |
| rs4672338 | 0.0085 | 0.0118 | 0.0081 | 0.0018 | 0.003 | 0.0117 |
| rs4673553 | 0.0056 | 0.0142 | 0.0079 | 0.0017 | 0.0129 | 0.0116 |
| rs4676084 | 0.0117 | 0.0098 | 0.0079 | 0.0017 | 0.0034 | 0.0117 |
| rs4677812 | -8.00E-04 | -0.0136 | 0.0087 | 0.0019 | -0.0038 | 0.0138 |
| rs4682718 | 0.0104 | 0.0154 | 0.0101 | 0.0023 | 0.0108 | 0.0138 |
| rs4683096 | -0.0274 | -0.0116 | 0.0078 | 0.0017 | -0.0152 | 0.0119 |
| rs4704513 | 0.0182 | 0.0125 | 0.01 | 0.0022 | 0.0091 | 0.0145 |
| rs4718966 | 0.0125 | 0.0127 | 0.008 | 0.0018 | 0.0245 | 0.0119 |
| rs4722398 | 0.0358 | 0.0158 | 0.0113 | 0.0025 | 0.0254 | 0.0231 |
| rs4740619 | -0.0169 | -0.0186 | 0.0074 | 0.0016 | -0.0029 | 0.0116 |
| rs4745794 | 0.0219 | 0.0114 | 0.0079 | 0.0017 | 0.0227 | 0.0117 |
| rs4757144 | 0.0163 | 0.0169 | 0.0079 | 0.0018 | 0.0338 | 0.0116 |
| rs4759075 | -0.019 | -0.0112 | 0.0074 | 0.0017 | -0.0221 | 0.0117 |
| rs4759228 | -0.0202 | -0.0157 | 0.0086 | 0.0019 | 0.0025 | 0.0136 |
| rs4766710 | -0.0085 | -0.0229 | 0.0159 | 0.0035 | -0.0277 | 0.0224 |
| rs4783830 | 0.0023 | -0.0105 | 0.0085 | 0.0019 | -0.0096 | 0.0124 |
| rs4812405 | -0.0429 | -0.0204 | 0.0154 | 0.0033 | -0.0179 | 0.0377 |
| rs4813619 | -0.0044 | -0.0108 | 0.0079 | 0.0018 | -0.0032 | 0.0117 |
| rs4820408 | -0.0198 | -0.0151 | 0.0074 | 0.0017 | 0.0046 | 0.0118 |
| rs4823182 | 0.0482 | -0.0012 | 0.0077 | 0.0017 | 0.0037 | 0.0117 |
| rs483752 | 0.0117 | 0.0121 | 0.0091 | 0.0022 | 0.0028 | 0.0135 |
| rs4841659 | 0.0346 | 0.0149 | 0.0085 | 0.0017 | -0.0372 | 0.0201 |
| rs4842491 | 0.0016 | 0.0098 | 0.008 | 0.0018 | 0.0042 | 0.0126 |
| rs4856794 | -0.0357 | 0.011 | 0.0083 | 0.0018 | -0.0204 | 0.0128 |
| rs4858193 | -0.0247 | -0.0129 | 0.0089 | 0.0019 | -0.0372 | 0.0132 |
| rs4858887 | 0.0159 | -0.0124 | 0.0083 | 0.0019 | 0.0116 | 0.0134 |
| rs4864201 | -0.013 | -0.0141 | 0.0075 | 0.0017 | -6.00E-04 | 0.012 |
| rs4865796 | 0.053 | -0.0103 | 0.0078 | 0.0018 | -0.0149 | 0.0123 |
| rs4877313 | -0.0117 | -0.0129 | 0.0095 | 0.0021 | -0.0093 | 0.0139 |
| rs488029 | 0.0344 | 0.0145 | 0.0079 | 0.0017 | 0.0046 | 0.0117 |
| rs4889606 | -0.0047 | -0.0202 | 0.0074 | 0.0017 | 0.0091 | 0.0119 |
| rs4906908 | 0.0042 | 0.0103 | 0.0078 | 0.0017 | 0.0057 | 0.0116 |
| rs4912637 | 0.0048 | -0.0103 | 0.0082 | 0.0018 | 0.0103 | 0.0124 |
| rs491711 | -0.0265 | -0.0115 | 0.0085 | 0.0019 | -0.0229 | 0.0138 |
| rs4929923 | 0.0205 | 0.0181 | 0.0074 | 0.0017 | 0.0054 | 0.0122 |
| rs4932143 | -0.0568 | 0.0049 | 0.0087 | 0.0019 | 0.0016 | 0.0133 |
| rs4954638 | -0.0021 | -0.0118 | 0.0091 | 0.002 | -0.004 | 0.0122 |
| rs4973925 | 0.0079 | 0.0107 | 0.0079 | 0.0017 | 0.0047 | 0.0117 |
| rs498240 | -0.0158 | -0.0267 | 0.0153 | 0.0033 | 0.036 | 0.0295 |
| rs4985155 | -0.0123 | -0.012 | 0.0076 | 0.0017 | -0.0133 | 0.0122 |
| rs516946 | 0.0824 | 2.00E-04 | 0.0085 | 0.0019 | -0.0148 | 0.0144 |
| rs5215 | -0.0678 | 0.0117 | 0.0073 | 0.0017 | 0.0192 | 0.0116 |
| rs538579 | 0.0246 | 0.0137 | 0.0084 | 0.0019 | 0.0149 | 0.0132 |
| rs543874 | 0.0346 | 0.0475 | 0.0089 | 0.002 | 0.0319 | 0.0152 |
| rs559231 | 0.0174 | 0.0135 | 0.008 | 0.0018 | 0.0012 | 0.0117 |
| rs576674 | -0.0654 | 0.0041 | 0.0097 | 0.0022 | 0.0183 | 0.0202 |
| rs577525 | 0.0101 | 0.0166 | 0.0078 | 0.0017 | 0.0061 | 0.0117 |
| rs591088 | -0.0038 | -0.0097 | 0.0078 | 0.0017 | -0.0142 | 0.0116 |
| rs6014523 | -0.0093 | -0.0149 | 0.0099 | 0.0022 | 0.0097 | 0.015 |
| rs6019482 | -0.0168 | -0.0178 | 0.0107 | 0.0023 | 9.00E-04 | 0.0156 |
| rs6023633 | -0.007 | -0.0138 | 0.0095 | 0.0021 | 0.0043 | 0.0149 |
| rs6050446 | 0.0459 | 0.0343 | 0.0213 | 0.0047 | 0.0534 | 0.0384 |
| rs6056413 | -0.0122 | -0.0127 | 0.0102 | 0.0022 | 0.0178 | 0.0141 |
| rs6066138 | -0.049 | 0.0016 | 0.0082 | 0.0018 | -0.0055 | 0.0142 |
| rs6121381 | 0.0195 | 0.0147 | 0.0107 | 0.0024 | -0.0103 | 0.0171 |
| rs621042 | -0.0075 | -0.0107 | 0.0079 | 0.0017 | -0.0019 | 0.0117 |
| rs6235 | -0.0048 | 0.0175 | 0.0088 | 0.0019 | 0.0155 | 0.0128 |
| rs6265 | -0.0151 | -0.0412 | 0.0092 | 0.0021 | -0.0058 | 0.016 |
| rs629443 | 0.0102 | -0.0116 | 0.0087 | 0.0019 | 0.0039 | 0.0132 |
| rs6419734 | -0.0129 | -0.0174 | 0.011 | 0.0025 | 0.0146 | 0.0173 |
| rs6433243 | -0.033 | -0.0102 | 0.0081 | 0.0018 | -0.0227 | 0.0125 |
| rs6435622 | 0.0216 | 0.0125 | 0.0084 | 0.0019 | 0.0012 | 0.0127 |
| rs6443750 | 0.027 | 0.0148 | 0.0096 | 0.0021 | -0.0019 | 0.0143 |
| rs6448587 | -0.013 | -0.0167 | 0.0103 | 0.0023 | -0.0137 | 0.0139 |
| rs6449532 | 0.0085 | -0.0127 | 0.0081 | 0.0018 | 0.0125 | 0.0128 |
| rs645482 | -0.0203 | -0.0109 | 0.0086 | 0.0019 | -0.0111 | 0.0126 |
| rs6456739 | 0.0058 | 0.0095 | 0.0078 | 0.0017 | 0.0126 | 0.0122 |
| rs6461115 | -0.0377 | -0.0144 | 0.0084 | 0.0019 | -0.01 | 0.0126 |
| rs6471941 | 0.0261 | 0.0156 | 0.0092 | 0.0021 | 0.0187 | 0.0124 |
| rs6477694 | -0.0139 | -0.0123 | 0.0074 | 0.0017 | -0.0287 | 0.0119 |
| rs6482729 | -0.001 | -0.0102 | 0.0083 | 0.0019 | -5.00E-04 | 0.0122 |
| rs6497676 | -0.0165 | -0.0098 | 0.0081 | 0.0018 | 0.0234 | 0.0125 |
| rs6515236 | -0.0504 | 0.0028 | 0.0091 | 0.002 | 0.0157 | 0.0143 |
| rs651548 | -0.0055 | -0.0139 | 0.0082 | 0.0018 | -2.00E-04 | 0.0121 |
| rs6545714 | -0.0383 | -0.0191 | 0.0074 | 0.0017 | 0.0028 | 0.0118 |
| rs6548220 | -0.0177 | -0.0131 | 0.0085 | 0.0019 | 0.0124 | 0.0132 |
| rs6564360 | 0.0133 | 0.0135 | 0.01 | 0.0022 | -0.031 | 0.0149 |
| rs6569648 | 0.031 | -0.0126 | 0.0084 | 0.0019 | -0.0014 | 0.0142 |
| rs657452 | 3.00E-04 | -0.0188 | 0.0075 | 0.0017 | 0.0012 | 0.0117 |
| rs6587552 | -0.0172 | -0.0173 | 0.0092 | 0.002 | -0.0125 | 0.013 |
| rs6593688 | 0.0165 | 0.0137 | 0.0081 | 0.0018 | 1.00E-04 | 0.0119 |
| rs6594967 | -0.02 | -0.0109 | 0.0084 | 0.0018 | 0.0058 | 0.0122 |
| rs659967 | 0.0366 | 0.0147 | 0.0113 | 0.0025 | -0.0066 | 0.0148 |
| rs6606686 | -0.0146 | -0.0136 | 0.0077 | 0.0018 | 0.0039 | 0.0124 |
| rs663129 | 0.0504 | 0.0545 | 0.0084 | 0.0019 | 0.0214 | 0.015 |
| rs6676084 | -0.0165 | -0.0122 | 0.0079 | 0.0018 | 0.0067 | 0.0125 |
| rs6690764 | -0.0318 | -0.0154 | 0.0095 | 0.0022 | -0.024 | 0.0139 |
| rs6692586 | -0.0132 | -0.0192 | 0.0104 | 0.0023 | -0.0033 | 0.0182 |
| rs6707445 | 0.0091 | 0.0128 | 0.008 | 0.0017 | -6.00E-04 | 0.0117 |
| rs6710871 | 0.0136 | 0.0179 | 0.011 | 0.0024 | -0.0246 | 0.0192 |
| rs6712 | 0.0411 | 0.0138 | 0.0118 | 0.0025 | -0.0227 | 0.0171 |
| rs6713781 | -0.0206 | -0.0123 | 0.0081 | 0.0018 | 0.0019 | 0.0124 |
| rs6738445 | 0.0108 | 0.0133 | 0.008 | 0.0018 | 0.0028 | 0.0141 |
| rs6764533 | 0.0243 | 0.0116 | 0.0082 | 0.0018 | 0.0091 | 0.0127 |
| rs6767619 | 0.005 | 0.0119 | 0.0083 | 0.0018 | 0.0201 | 0.0126 |
| rs6772756 | -0.0302 | -0.0104 | 0.0085 | 0.0019 | 0.0014 | 0.0122 |
| rs6781254 | 0.0096 | 0.0112 | 0.008 | 0.0018 | -0.0195 | 0.0127 |
| rs6795735 | -0.0558 | 0.0059 | 0.0073 | 0.0017 | 0.01 | 0.0121 |
| rs6804181 | -0.0111 | -0.0153 | 0.0103 | 0.0023 | 0.0597 | 0.0139 |
| rs6804842 | 0.0207 | 0.0156 | 0.0074 | 0.0017 | -0.0142 | 0.0116 |
| rs6808574 | 0.0552 | 5.00E-04 | 0.0076 | 0.0017 | -6.00E-04 | 0.0119 |
| rs6818414 | 0.0042 | 0.0097 | 0.0079 | 0.0017 | 0.0152 | 0.0116 |
| rs6841761 | -0.0195 | -0.0131 | 0.0072 | 0.0016 | -0.0071 | 0.0119 |
| rs6849518 | 0.0497 | 0.0173 | 0.0115 | 0.0026 | 0.0112 | 0.0155 |
| rs6850639 | 0.0251 | 0.0124 | 0.0091 | 0.0021 | 0.0094 | 0.0158 |
| rs6852276 | -0.0063 | 0.0108 | 0.0074 | 0.0017 | 0.0116 | 0.0121 |
| rs6864049 | 0.0033 | 0.0125 | 0.0072 | 0.0017 | 0.0134 | 0.0118 |
| rs6879326 | 0.0141 | 0.0098 | 0.0078 | 0.0017 | -0.0039 | 0.0116 |
| rs6950442 | 0.0216 | 0.0165 | 0.0097 | 0.0022 | 0.0031 | 0.0137 |
| rs6963840 | 0.0213 | 0.0154 | 0.0107 | 0.0024 | -0.0199 | 0.0185 |
| rs6968554 | 0.0195 | 0.01 | 0.0076 | 0.0017 | -0.0075 | 0.0124 |
| rs6985109 | -0.0422 | -0.0177 | 0.0076 | 0.0017 | -0.0193 | 0.019 |
| rs7006629 | 0.0044 | 0.0109 | 0.0078 | 0.0017 | -0.0023 | 0.0117 |
| rs7024334 | -0.0128 | -0.0138 | 0.0087 | 0.002 | -0.0237 | 0.0142 |
| rs7025938 | 0.0134 | 0.0166 | 0.0084 | 0.0019 | -0.0011 | 0.0119 |
| rs702820 | 0.0108 | 0.017 | 0.0121 | 0.0027 | 0.0406 | 0.0182 |
| rs7031064 | -0.0113 | -0.0108 | 0.0078 | 0.0017 | -0.0072 | 0.0117 |
| rs7042372 | -2.00E-04 | -0.0122 | 0.0083 | 0.0018 | -0.0033 | 0.0119 |
| rs705980 | -0.0242 | -0.0112 | 0.0079 | 0.0017 | -0.003 | 0.0118 |
| rs7075281 | 0.0014 | -0.0121 | 0.0078 | 0.0018 | -0.008 | 0.0133 |
| rs7084454 | -0.0013 | 0.0193 | 0.0083 | 0.0019 | 0.02 | 0.0126 |
| rs709400 | -0.0217 | -0.015 | 0.0075 | 0.0017 | -0.0049 | 0.0126 |
| rs7102454 | -0.0012 | 0.0158 | 0.0081 | 0.0018 | -0.0116 | 0.0129 |
| rs7117238 | -0.0123 | -0.0131 | 0.0097 | 0.0022 | 0.0016 | 0.0146 |
| rs7117842 | 0.001 | 0.0111 | 0.0075 | 0.0017 | -0.0109 | 0.012 |
| rs7120873 | 0.0463 | 0.0172 | 0.0126 | 0.0027 | 0.019 | 0.0218 |
| rs7124681 | 0.0369 | 0.0263 | 0.0073 | 0.0016 | -0.0102 | 0.0122 |
| rs7134628 | 0.0087 | 0.0172 | 0.0127 | 0.0028 | 0.0052 | 0.0206 |
| rs7138300 | -0.0443 | 2.00E-04 | 0.0072 | 0.0016 | -0.0014 | 0.012 |
| rs7138803 | 0.0336 | 0.03 | 0.0073 | 0.0017 | 0.0077 | 0.012 |
| rs7144011 | 0.0482 | 0.0282 | 0.0085 | 0.002 | 0.0175 | 0.0136 |
| rs7164727 | 0.0173 | 0.0182 | 0.0077 | 0.0017 | 0.0071 | 0.0125 |
| rs716764 | -0.0101 | -0.0179 | 0.0104 | 0.0023 | -0.0245 | 0.0158 |
| rs7172627 | 0.0124 | 0.0117 | 0.0078 | 0.0017 | 0.006 | 0.0116 |
| rs7177055 | 0.0647 | 0.0091 | 0.0079 | 0.0018 | 0.0147 | 0.0126 |
| rs7181498 | -0.0253 | -0.0163 | 0.0082 | 0.0018 | 0.0178 | 0.0117 |
| rs7195386 | 0.008 | -0.0133 | 0.0079 | 0.0017 | 0.0071 | 0.0116 |
| rs719802 | -0.0224 | -0.0101 | 0.008 | 0.0018 | 0.0047 | 0.0123 |
| rs7200395 | 0.0106 | 0.0133 | 0.0083 | 0.0019 | -0.0052 | 0.0129 |
| rs7200919 | 0.0069 | 0.0095 | 0.0073 | 0.0017 | 1.00E-04 | 0.0117 |
| rs7206395 | 0.0149 | 0.0123 | 0.0099 | 0.0022 | 0.0173 | 0.0177 |
| rs7206608 | 0.0222 | 0.0132 | 0.0083 | 0.0019 | 0.0397 | 0.0121 |
| rs7209235 | -0.0027 | -0.0111 | 0.0086 | 0.0019 | -0.0026 | 0.0139 |
| rs7220138 | 2.00E-04 | 0.0123 | 0.0086 | 0.0019 | -0.0068 | 0.0124 |
| rs7235205 | 0.028 | 0.0141 | 0.0081 | 0.0019 | -4.00E-04 | 0.0119 |
| rs7235563 | -0.0129 | -0.0098 | 0.0082 | 0.0018 | -0.0124 | 0.012 |
| rs7239114 | 0.0121 | 0.0124 | 0.0074 | 0.0017 | 0.0295 | 0.0117 |
| rs7239575 | -0.0331 | -0.0202 | 0.0078 | 0.0017 | -0.0164 | 0.0116 |
| rs7249143 | 0.0322 | 0.0126 | 0.0086 | 0.0019 | 8.00E-04 | 0.0121 |
| rs731834 | -0.0018 | -0.0107 | 0.0079 | 0.0018 | 0.0183 | 0.0118 |
| rs7318817 | -0.0264 | -0.0155 | 0.0081 | 0.0018 | -0.033 | 0.0119 |
| rs7323827 | -0.0044 | -0.0102 | 0.0083 | 0.0018 | 0.0141 | 0.0128 |
| rs7332115 | -0.0281 | -0.0159 | 0.0075 | 0.0017 | 0.0141 | 0.0117 |
| rs7333719 | -0.016 | -0.0127 | 0.0104 | 0.0023 | -0.0082 | 0.0152 |
| rs7334078 | -0.013 | -0.0121 | 0.0086 | 0.0019 | -1.00E-04 | 0.0134 |
| rs733594 | 0.016 | 0.0138 | 0.0081 | 0.0018 | -0.0149 | 0.0131 |
| rs735949 | -0.0711 | 0.0031 | 0.0106 | 0.0024 | -0.0041 | 0.0194 |
| rs738140 | -0.0315 | -0.0135 | 0.0086 | 0.0019 | 0.0033 | 0.0124 |
| rs740158 | -0.0259 | -0.0114 | 0.0073 | 0.0016 | -0.0298 | 0.0116 |
| rs7488867 | -0.0285 | -0.0204 | 0.009 | 0.002 | 0.0141 | 0.0126 |
| rs7498665 | 0.0173 | 0.0271 | 0.007 | 0.0017 | -0.023 | 0.0118 |
| rs7519259 | 0.0183 | 0.0125 | 0.0079 | 0.0017 | 0.0074 | 0.0117 |
| rs7526754 | -0.0171 | -0.0103 | 0.0083 | 0.0018 | -0.0065 | 0.0119 |
| rs753270 | 0.0528 | 0.0049 | 0.0079 | 0.0018 | -0.0169 | 0.0118 |
| rs7534091 | 0.0159 | 0.0113 | 0.0081 | 0.0019 | 0.0069 | 0.0136 |
| rs7551507 | -0.0055 | -0.0184 | 0.0072 | 0.0016 | -0.0102 | 0.0116 |
| rs7557796 | -0.0203 | -0.016 | 0.0082 | 0.0018 | -8.00E-04 | 0.0119 |
| rs7560871 | 0.0204 | 0.0218 | 0.0151 | 0.0034 | 0.006 | 0.0224 |
| rs7561278 | 0.0032 | -0.0159 | 0.0095 | 0.0021 | 0.0144 | 0.0137 |
| rs7567655 | 0.0564 | 0.027 | 0.0189 | 0.0045 | -0.0356 | 0.0264 |
| rs7572970 | 0.059 | 0.0093 | 0.0087 | 0.0019 | 0.0111 | 0.0145 |
| rs7599312 | -9.00E-04 | -0.0186 | 0.0081 | 0.0019 | -0.0054 | 0.0127 |
| rs7600699 | -0.0018 | 0.0149 | 0.0108 | 0.0024 | -0.0026 | 0.0176 |
| rs7601895 | -0.0141 | -0.0149 | 0.0086 | 0.0019 | 0.0033 | 0.0127 |
| rs7607351 | -0.0066 | -0.0119 | 0.008 | 0.0017 | -0.0484 | 0.0119 |
| rs7615297 | -0.0318 | -0.0149 | 0.0109 | 0.0024 | 0.0142 | 0.0157 |
| rs762147 | -3.00E-04 | 0.0115 | 0.0088 | 0.0019 | -0.0046 | 0.0128 |
| rs7630080 | -0.0151 | -0.0168 | 0.0138 | 0.0031 | 0.013 | 0.0297 |
| rs7640424 | -0.0277 | -0.0136 | 0.0081 | 0.0018 | -0.0075 | 0.0121 |
| rs7651090 | 0.1204 | -0.006 | 0.0076 | 0.0018 | 0.0103 | 0.0126 |
| rs7652415 | 0.0217 | 0.0156 | 0.0114 | 0.0026 | 0.0125 | 0.0191 |
| rs7655341 | 0.0302 | 0.0115 | 0.0088 | 0.0019 | 0.0117 | 0.0135 |
| rs765875 | -0.0156 | -0.0121 | 0.0078 | 0.0017 | 0.0015 | 0.0118 |
| rs7674623 | 0.024 | 0.0135 | 0.0098 | 0.0022 | 0.0062 | 0.0156 |
| rs7683836 | -0.0104 | -0.0114 | 0.0079 | 0.0017 | 0.0089 | 0.0116 |
| rs7685048 | -0.0348 | -0.0101 | 0.0079 | 0.0017 | 0.0019 | 0.0119 |
| rs7685296 | -0.0511 | 0.0021 | 0.0081 | 0.0018 | -0.0049 | 0.0128 |
| rs7694732 | -0.0045 | -0.0099 | 0.0079 | 0.0017 | 0.0053 | 0.0117 |
| rs769674 | -0.0335 | -0.0135 | 0.0084 | 0.0018 | -0.0149 | 0.0123 |
| rs7715256 | -0.0166 | -0.0166 | 0.0073 | 0.0016 | 0.0091 | 0.0118 |
| rs7716275 | -0.0159 | -0.0132 | 0.0092 | 0.0021 | 0.0167 | 0.0151 |
| rs77258096 | -0.1171 | 0.0052 | 0.0134 | 0.0025 | -0.0376 | 0.0196 |
| rs7729395 | 0.1373 | 0.0051 | 0.016 | 0.0038 | -0.0086 | 0.0253 |
| rs7730004 | 0.0086 | 0.0148 | 0.0084 | 0.0018 | -0.0221 | 0.0117 |
| rs7730898 | 0.0186 | 0.0168 | 0.0081 | 0.0018 | 0.0298 | 0.0124 |
| rs7756992 | 0.1297 | -0.0089 | 0.0078 | 0.0018 | -0.0222 | 0.0123 |
| rs7760082 | -0.0152 | -0.0122 | 0.0083 | 0.0018 | -0.0123 | 0.0122 |
| rs7761673 | -0.0282 | -0.0126 | 0.0093 | 0.0021 | -0.0044 | 0.0163 |
| rs7780752 | -0.0015 | 0.0139 | 0.0081 | 0.0018 | 0.0152 | 0.0132 |
| rs7784465 | 6.00E-04 | 0.0164 | 0.0115 | 0.0025 | 0.0185 | 0.0168 |
| rs7786095 | -0.0743 | -0.003 | 0.0129 | 0.0028 | -0.0156 | 0.0248 |
| rs779206 | -0.0011 | -0.0127 | 0.0089 | 0.002 | -0.0172 | 0.0144 |
| rs7802342 | 0.0126 | 0.0121 | 0.0086 | 0.0019 | -0.0045 | 0.0132 |
| rs7811342 | 0.0184 | -0.0197 | 0.0129 | 0.0029 | -0.0029 | 0.0154 |
| rs7826312 | 0.0085 | 0.0104 | 0.0074 | 0.0017 | -0.0024 | 0.0117 |
| rs7844647 | -0.0087 | -0.0123 | 0.0082 | 0.0018 | -9.00E-04 | 0.0135 |
| rs784944 | -0.0361 | -0.0122 | 0.0088 | 0.0019 | -0.0049 | 0.0129 |
| rs7865157 | 0.0167 | 0.0177 | 0.0128 | 0.0028 | 0.0248 | 0.0212 |
| rs7869771 | 0.0028 | -0.014 | 0.0087 | 0.0019 | 0.0021 | 0.0143 |
| rs7899106 | 0.0279 | 0.0331 | 0.0164 | 0.0037 | 0.0435 | 0.0281 |
| rs7903146 | 0.3059 | -0.0181 | 0.0077 | 0.0018 | 0.0252 | 0.0146 |
| rs7923866 | -0.0972 | 0.0054 | 0.0074 | 0.0017 | -0.0071 | 0.0118 |
| rs7925214 | 0.0185 | 0.0147 | 0.0079 | 0.0018 | 0.0118 | 0.0117 |
| rs7929418 | -0.0175 | -0.0113 | 0.008 | 0.0018 | -0.0028 | 0.012 |
| rs7933205 | 0.0138 | 0.0116 | 0.0095 | 0.0021 | 4.00E-04 | 0.0161 |
| rs7947143 | 0.0061 | -0.018 | 0.0109 | 0.0024 | 0.0023 | 0.0159 |
| rs7970953 | 0.0214 | 0.0135 | 0.0078 | 0.0018 | 0.0148 | 0.0126 |
| rs7973955 | -0.0251 | -0.0126 | 0.0087 | 0.0019 | -0.0298 | 0.013 |
| rs8016771 | 0.0451 | 0.019 | 0.0137 | 0.0031 | 0.021 | 0.0199 |
| rs802460 | 0.014 | 0.0107 | 0.0082 | 0.0018 | -0.023 | 0.0119 |
| rs8033995 | -0.028 | -0.0166 | 0.0133 | 0.0029 | 0.0023 | 0.0219 |
| rs8036040 | 0.0102 | 0.0109 | 0.0078 | 0.0017 | -0.0014 | 0.0116 |
| rs8047395 | 0.0884 | 0.0642 | 0.0073 | 0.0017 | 0.0118 | 0.0116 |
| rs8060878 | 0.0225 | 0.0112 | 0.0072 | 0.0016 | -0.0041 | 0.0116 |
| rs8068804 | 0.0587 | 0.0085 | 0.0078 | 0.0018 | -0.0024 | 0.0127 |
| rs8070454 | -0.002 | -0.0098 | 0.0074 | 0.0017 | -0.0232 | 0.0119 |
| rs8071182 | 0.0116 | 0.0133 | 0.0095 | 0.0022 | 0.0053 | 0.0161 |
| rs8075273 | -0.0404 | -0.0133 | 0.0081 | 0.0018 | -0.0011 | 0.0139 |
| rs8092503 | 0.0118 | 0.0165 | 0.0084 | 0.0019 | -0.012 | 0.0123 |
| rs8097672 | 0.0467 | 0.02 | 0.011 | 0.0025 | 0.0401 | 0.0153 |
| rs8097783 | -0.0178 | -0.0389 | 0.014 | 0.0031 | -0.0234 | 0.0294 |
| rs8123881 | 0.0113 | 0.0196 | 0.0106 | 0.0024 | -0.001 | 0.0136 |
| rs8181823 | 0.0243 | 0.0127 | 0.0091 | 0.002 | 0.0174 | 0.0131 |
| rs818524 | -0.0038 | 0.0106 | 0.0086 | 0.0019 | 0.0056 | 0.0132 |
| rs8192675 | -0.0439 | 0.0152 | 0.0079 | 0.0018 | 0.0075 | 0.0131 |
| rs823074 | -0.0172 | -0.0112 | 0.008 | 0.0017 | -0.0012 | 0.0117 |
| rs825476 | 0.0524 | -0.0095 | 0.0073 | 0.0016 | 0.0084 | 0.0118 |
| rs825680 | -0.0124 | -0.0104 | 0.008 | 0.0018 | -0.0148 | 0.0119 |
| rs845084 | 0.0234 | 0.014 | 0.0088 | 0.002 | 0.0035 | 0.0133 |
| rs849135 | -0.0999 | 0.0109 | 0.0072 | 0.0016 | -0.0533 | 0.0117 |
| rs852056 | -0.0079 | -0.0128 | 0.0087 | 0.002 | 0.0263 | 0.0137 |
| rs853681 | 0.0241 | 0.0143 | 0.0112 | 0.0024 | 0.034 | 0.0231 |
| rs857601 | 0.0208 | 0.0104 | 0.0088 | 0.0019 | 0.0078 | 0.0128 |
| rs881301 | 0.0145 | 0.0097 | 0.0079 | 0.0017 | -0.0162 | 0.0117 |
| rs889398 | -0.0411 | -0.0196 | 0.0073 | 0.0016 | -0.0176 | 0.0118 |
| rs896183 | 0.0032 | 0.0102 | 0.0074 | 0.0017 | -0.0043 | 0.0119 |
| rs901630 | -0.0238 | -0.0146 | 0.0073 | 0.0017 | 0.0066 | 0.0123 |
| rs903959 | 0.0117 | 0.0106 | 0.008 | 0.0018 | -0.0086 | 0.012 |
| rs925421 | -0.0092 | -0.0116 | 0.0088 | 0.002 | -0.021 | 0.0131 |
| rs9294260 | 0.0181 | 0.0147 | 0.0076 | 0.0016 | 0.0113 | 0.0116 |
| rs9296723 | 0.0044 | 0.0106 | 0.0076 | 0.0018 | -0.0021 | 0.0133 |
| rs930295 | -0.0207 | -0.0211 | 0.0105 | 0.0023 | 0.0138 | 0.0173 |
| rs9321951 | 0.0114 | 0.0102 | 0.0084 | 0.0019 | 0.0145 | 0.0126 |
| rs9326846 | 0.017 | 0.0108 | 0.0084 | 0.0019 | 0.0022 | 0.012 |
| rs934515 | 0.0203 | 0.0185 | 0.0123 | 0.0027 | 2.00E-04 | 0.0153 |
| rs9349239 | -0.0074 | -0.0122 | 0.0072 | 0.0017 | 0.0339 | 0.0117 |
| rs9361779 | 0.0321 | 0.011 | 0.0079 | 0.0017 | 0.0154 | 0.0119 |
| rs936227 | 0.0045 | 0.0118 | 0.0074 | 0.0017 | 0.0016 | 0.0117 |
| rs9362662 | -0.0012 | -0.0112 | 0.0079 | 0.0017 | -0.0058 | 0.0117 |
| rs9364687 | 0.0021 | -0.0106 | 0.0072 | 0.0017 | -0.0181 | 0.0116 |
| rs9367368 | -0.0233 | -0.0121 | 0.0078 | 0.0018 | -0.0068 | 0.0119 |
| rs9397927 | -0.0013 | 0.0119 | 0.009 | 0.002 | 0.0013 | 0.0121 |
| rs9408882 | -0.004 | -0.0093 | 0.0074 | 0.0016 | -0.0155 | 0.0116 |
| rs9426003 | -0.0138 | -0.0116 | 0.0087 | 0.0019 | 0.0217 | 0.0134 |
| rs9436446 | 0.009 | 0.0171 | 0.0093 | 0.002 | 0.0045 | 0.0142 |
| rs9463175 | -0.0021 | -0.0108 | 0.0078 | 0.0017 | -0.0235 | 0.0123 |
| rs946526 | 0.0642 | 0.0314 | 0.0198 | 0.0043 | 0.0672 | 0.0343 |
| rs946824 | -0.027 | -0.0206 | 0.0116 | 0.0026 | -0.0035 | 0.0164 |
| rs9475173 | -0.0025 | 0.0108 | 0.0083 | 0.0019 | 0.0279 | 0.0117 |
| rs947612 | -0.0129 | -0.0116 | 0.009 | 0.002 | -0.0256 | 0.013 |
| rs9478496 | 0.0233 | 0.0152 | 0.0105 | 0.0023 | 0.0343 | 0.0145 |
| rs9522285 | 0.0293 | 0.0127 | 0.0079 | 0.0017 | 0.0251 | 0.0116 |
| rs9527455 | -0.0243 | -0.0117 | 0.0093 | 0.002 | -0.0045 | 0.0136 |
| rs9527958 | 0.0143 | 0.0102 | 0.0082 | 0.0018 | 0.0084 | 0.0127 |
| rs9530843 | -0.0033 | -0.0128 | 0.0073 | 0.0018 | 4.00E-04 | 0.0124 |
| rs9538141 | 0.0108 | 0.0164 | 0.0078 | 0.0017 | -0.0182 | 0.0117 |
| rs954018 | 0.0158 | -0.013 | 0.0078 | 0.0018 | -0.0015 | 0.0121 |
| rs9540493 | -0.0304 | -0.0139 | 0.0072 | 0.0017 | -0.0297 | 0.0117 |
| rs9547153 | 0.0064 | 0.0098 | 0.0075 | 0.0017 | 0.0068 | 0.0119 |
| rs9571687 | -0.0173 | -0.0129 | 0.0084 | 0.0018 | -0.0069 | 0.0139 |
| rs961917 | -0.0091 | -0.0116 | 0.0088 | 0.002 | -0.0072 | 0.0134 |
| rs962796 | -0.0261 | -0.0148 | 0.0096 | 0.0022 | -0.0296 | 0.0146 |
| rs963740 | -0.0479 | 0.0014 | 0.0086 | 0.0019 | 0.0055 | 0.0143 |
| rs9650755 | 0.0287 | 0.0154 | 0.0088 | 0.002 | 0.0215 | 0.0126 |
| rs9688431 | -0.0191 | -0.0231 | 0.0155 | 0.0035 | -0.0581 | 0.0342 |
| rs977747 | -0.0238 | -0.0169 | 0.0073 | 0.0017 | 0.0074 | 0.0121 |
| rs9787495 | -0.0199 | -0.0103 | 0.0079 | 0.0017 | -0.0123 | 0.012 |
| rs9806058 | -0.0222 | -0.0164 | 0.0119 | 0.0026 | 0.0165 | 0.0214 |
| rs9816226 | 0.0351 | 0.0323 | 0.0094 | 0.0021 | 0.008 | 0.0158 |
| rs9827823 | -0.0357 | -0.0193 | 0.0111 | 0.0024 | -0.0243 | 0.018 |
| rs9844972 | 0.0956 | -0.0012 | 0.0148 | 0.0035 | -0.0069 | 0.0256 |
| rs9845966 | -0.0289 | -0.0105 | 0.0074 | 0.0017 | 0.0056 | 0.0116 |
| rs9848399 | -0.0045 | -0.0164 | 0.0117 | 0.0026 | -0.0094 | 0.0154 |
| rs987237 | 0.0586 | 0.0409 | 0.009 | 0.0021 | 0.0107 | 0.0142 |
| rs9894220 | -0.0585 | -0.01 | 0.0079 | 0.0018 | 0.0096 | 0.0118 |
| rs9927848 | -0.0153 | -0.0122 | 0.0089 | 0.002 | -0.0088 | 0.0146 |
| rs993380 | -0.0507 | 0.0017 | 0.0081 | 0.0018 | -0.0149 | 0.0118 |
| rs9951619 | 0.0398 | 0.0156 | 0.0084 | 0.002 | -0.0073 | 0.0124 |
| rs9964756 | 0.0251 | 0.0164 | 0.0126 | 0.0028 | 0.0282 | 0.0173 |
| rs9965170 | -0.0136 | -0.0097 | 0.0078 | 0.0017 | 0.0088 | 0.0116 |
| rs998732 | -0.012 | -0.0171 | 0.01 | 0.0022 | -0.0308 | 0.0194 |
| rs9989141 | 0.0101 | 0.0162 | 0.0081 | 0.0017 | 0.0115 | 0.012 |
| rs999889 | -0.0161 | -0.0108 | 0.0088 | 0.0019 | -0.0105 | 0.0136 |

# Supplementary Figure


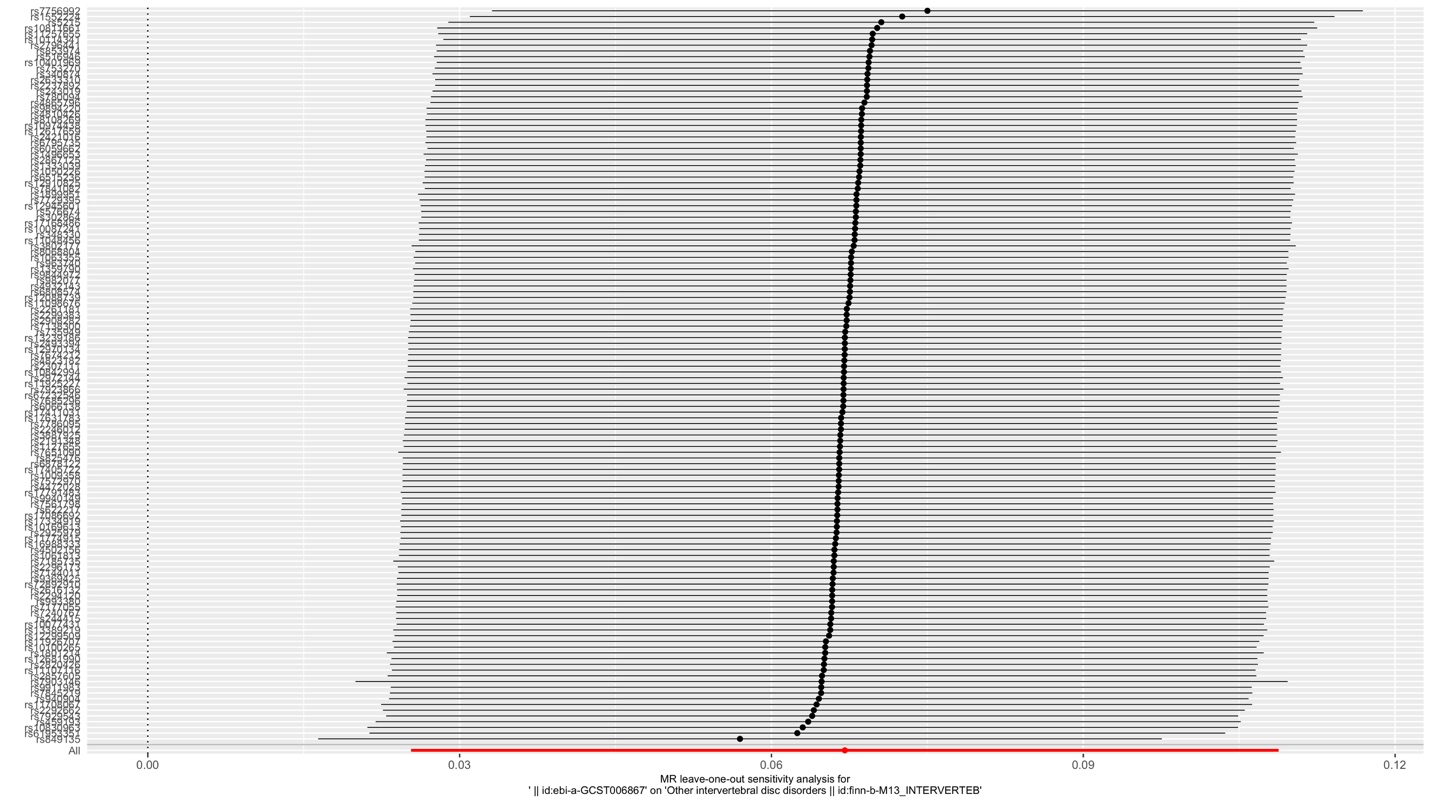


Supplementary Figure 1. MR leave-one-out sensitivity analysis for T2DM on IVDD
